# Investigating Cultural Evolution Using Phylogenetic Analysis: The Origins and Descent of the Southeast Asian Tradition of Warp Ikat Weaving

**DOI:** 10.1371/journal.pone.0052064

**Published:** 2012-12-18

**Authors:** Christopher D. Buckley

**Affiliations:** Independent Researcher, Beijing, People’s Republic of China; Durham University, United Kingdom

## Abstract

The warp ikat method of making decorated textiles is one of the most geographically widespread in southeast Asia, being used by Austronesian peoples in Indonesia, Malaysia and the Philippines, and Daic peoples on the Asian mainland. In this study a dataset consisting of the decorative characters of 36 of these warp ikat weaving traditions is investigated using Bayesian and Neighbornet techniques, and the results are used to construct a phylogenetic tree and taxonomy for warp ikat weaving in southeast Asia. The results and analysis show that these diverse traditions have a common ancestor amongst neolithic cultures the Asian mainland, and parallels exist between the patterns of textile weaving descent and linguistic phylogeny for the Austronesian group. Ancestral state analysis is used to reconstruct some of the features of the ancestral weaving tradition. The widely held theory that weaving motifs originated in the late Bronze Age Dong-Son culture is shown to be inconsistent with the data.

## Introduction

Southeast Asia possesses diverse textile weaving traditions, some of the most impressive achievements of which are represented in public and private collections worldwide and which have recently been the subject of several major exhibitions and publications [1]–[3]. These textiles have come to be recognized for the role they play in the cultural life of the region as markers of status, as decorative hangings to demarcate ritual spaces, in ritual gift exchanges on marriage, in rites of passage relating to birth and death, and as artistic achievements in their own right [4]–[10]. A variety of techniques are used to make decorated textiles, which include ikat, warp patterning, weft patterning, batik and embroidery. Of these, the first two (ikat and warp patterning) are believed to have a long history in the region [7], [9]. This view is based on several factors: these techniques are geographically widespread, being present in virtually all parts of Southeast Asia (SEA); they require only indigenous raw materials for their application, and they retain some features that are presumed to have arisen at a relatively early date, even though no precise chronology of weaving technologies exists. For example, warp ikat (together with supplementary warp patterning) was, until recently, still made using locally sourced bast and leaf fibers in a few areas, notably Hainan Island, Mindanao and Tanimbar. These materials were the main source of fiber for textile making before the advent of cotton and silk to the region.

Textile scholars have long noted similarities between ikat weaving techniques and motifs between different parts of SEA and speculated that they are connected or share a common origin [3], [4], [7], [9], [11], [12]. Several authors have put forward views on the origins of the weaving traditions of SEA, with most identifying the Asian mainland as the ultimate source. Within this consensus there are two main schools of thought: one school can be traced to the writings of Heine-Geldern, who pointed out similarities between Bronze Age cultures on the Asian mainland and artifacts including carving, weaving and other material culture from Island Southeast Asia (ISEA). Based on these similarities he postulated a Bronze Age mainland source for a substantial part of ISEA decorative art [13]. In a later publication, Heine-Geldern refined this theory to focus on the late Bronze Age Dong-Son culture that existed in a coastal area of what is now Vietnam, between 500BC and 100AC approximately [14]. He based his reasoning on the distribution of bronze drums made by Dong-Son people, which were exported across a wide area of what is now the Indonesian archipelago and which played an important role in the ritual life of many ISEA cultures. He postulated that both the technology of warp ikat weaving and the decorative motifs that it uses originated with the Dong-Son culture. This theory subsequently gained wide circulation in the literature relating to textiles and beyond, and has become an accepted explanation in some circles for the origins of many aspects of ISEA culture. An alternative view has been put forward more recently by some scholars who have pointed out the resemblances between SEA textile designs and neolithic pottery motifs [3], [4], [5], suggesting that the origin of these traditions may in fact lie in earlier cultures.

In traditional societies the main route for the transmission of culture, consisting of information and skills, is from older generations (parents and grandparents) to younger, by word-of-mouth and by practical demonstration. From time to time modifications and improvements are made, while some older aspects may fall into disuse and be forgotten. This form of transmission of information is a powerful and defining feature of human societies. It allowed pre-modern societies to develop complex skills and artifacts, including language, tool use and artistic traditions, and to refine and elaborate them over many generations, in spite of the absence of written records. In the case of weaving this transmission takes place mainly from mother to daughter, and from other female relatives and individuals in the close community. Older examples of textiles also serve as models for younger weavers to copy or adapt. Traditional textile motifs have importance as markers of familial or cultural affiliation and tend to be transmitted conservatively. For example, in his study of warp ikat weavers from the Sikka regency in Flores, Lewis [15] wrote: “The motifs of the cloths themselves belong to and are transmitted by lineally related women. Women pass onto their daughters, sister’s daughters, and in some cases, their brother’s daughters, particular motifs and the right to weave them.” This is especially true of the most elaborate cloths with important social functions (called adat cloths by Indonesian weavers), which are also the richest repositories of motifs. In her study of Lamaholot ikat cloth Barnes [4] wrote: “The adat cloth is supposed to be strictly traditional: only the patterns which have ‘always’ been used locally are permitted to appear in the ikat decoration.”

A further important feature of ikat, and indeed of most traditional textiles, is that weavers tend to use a narrow repertoire of simple shapes such as dashes, hooks and curls, in a ‘building block’ fashion to create their designs. This approach makes understanding and copying complex designs manageable for the weaver, and aids their transmission from generation to generation. Recognition of this repertoire of basic shapes (which I call primitives) is also of considerable help to the researcher with analyzing and comparing motifs between traditions, as discussed in the Materials and Methods section.

While weaving motifs and their building blocks are transmitted in a conservative fashion, the weaver may have considerable discretion in the arrangement of motifs and the layout of the overall cloth. It is notable that in cases where ‘new designs’ have been produced within living memory the novel elements usually relate to the layout of the cloth, rather than the motifs, such as the influential designs created by the late Theresia Sue of the Ndona district of Flores, discussed by Hamilton [16]. Such fashions may spread rapidly through a weaving community, while the underlying repertoire of motifs is more resistant to change.

Despite the conservatism of traditional weaving, copying of designs does occasionally occur. For example, designs associated with high status in one social group may be copied by another. Imported luxury textiles, particularly those from India, have been another source of copied motifs amongst Indonesian weavers. When copying of a design from a foreign source, weavers are likely to re-interpret the copied motif in terms of their own vocabulary of basic shapes rather than produce an exact copy. This is analogous to linguistic borrowing, where the pronunciation of a borrowed word may change from one language to another according to the repertoire of sounds available. An example of this phenomenon that shows how Biboki weavers in West Timor have rendered a borrowed Indian motif within the confines of their own set of basic shapes is discussed in Motifs S1. Instances of borrowing from distant sources are usually relatively easy to spot, but borrowing from neighboring groups that use a similar basic repertoire of shapes is more difficult to detect.

Transmission from one generation to the next with minor changes is called ‘descent with modification’, and is a familiar and fundamental one for biologists. Its importance to the existence of and relationship between biological species was first recognized by Darwin. During the middle part of the 20^th^ century, the underlying logic of this process was formalized by biologists into the discipline termed cladistics. This assumes that species (taxa) trace their descent from a common ancestor and that this descent can be depicted by a phylogenetic tree. New species are formed when existing species become geographically separated and drift apart through the cumulative effects of the descent with modification process. All other things being equal, species that share more characteristics can be expected to have diverged more recently. This principle allows potential phylogenetic trees to be constructed and tested against lists of the observed characteristics of species. These ideas were first applied to the physical characteristics (morphology) of organisms, and more recently to the genetic code itself. Darwin’s ideas are popularly associated with the idea of competition between species, but an important feature of the cladistic model is that competition is not a requirement: the process of descent with modification (giving rise to small, cumulative changes) is sufficient in itself to differentiate taxa and produce a phylogenetic tree.

Because traditional human culture is transmitted by a similar process of descent with modification, it has been recognized in recent decades that it may also be amenable to phylogenetic analysis. Linguists were amongst the first to explore this possibility, and phylogenetic analysis of linguistic data is now yielding important insights into the evolution of languages. There is an ongoing and informative dialogue between the practitioners of classical comparative methods and phylogenetic methods, particularly relating to the ISEA region [17]–[21]. A growing field also exists of applying phylogenetic methods to material culture, which has been the subject of several recent reviews and publications [22]–[25]. Within this field, a series of studies by Collard, Shennan, Tehrani and co-workers [26]–[29] has pioneered the use of textile motifs to reconstruct the phylogeny of carpet weaving amongst Turkmen tribes and to reveal the relationships between them. In these cultural studies the equivalent of a biological species or ‘taxon’ is the culture or language under consideration. Relevant characters (characteristics) of taxa that are analogous to morphological characteristics of biological species are given definitions and coded. Whether derived from biology, linguistics or material culture, the quality of a given dataset and its ability to reveal phylogenetic information depends upon heritable characters of the taxa being correctly identified and coded.

In phylogenetics/cladistic analysis the concept of a ‘clade’ is particularly important. In this paper I will use this term to mean a set of taxa (in this case, weaving traditions) that form one branch of a phylogenetic tree, in other words share a common ancestor. I will use the similar but more general term ‘group’ to denote a set of weaving traditions or languages (for example) that are believed to be related, but where the relationship was not necessarily deduced by phylogenetic means.

A major challenge faced by phylogenetic methods, whether in biological or cultural fields, is the gradual loss of the phylogenetic ‘signal’ that occurs over time, through random drift or through ‘horizontal transfer’. The process of random drift is a fundamental consequence of the descent with modification process, and means that over a large enough number of generations the original characteristics will eventually be lost or become too few to detect. Horizontal transfer refers to information that is acquired from external sources, as opposed to ‘vertical’ transfer from one generation to the next. In biology the process of sexual reproduction and hybridization (in higher organisms) and DNA exchange (in bacteria) complicate phylogenies by introducing genetic material from outside and making lineages into net-like structures rather than neat family trees. In linguistics and culture, the process of borrowing or copying ideas, words, technologies and designs from outside the village or cultural group has a similar effect. These effects may degrade or obscure an underlying phylogenetic signal. Nevertheless, studies show that a phylogenetic tree can still be recovered under conditions where horizontal transmission has taken place [30], if the phylogenetic component has not been completely lost.

Because of the complexity involved with large datasets and the large number of potential phylogenetic trees that must be tested, contemporary studies use computer-based methods to investigate phylogenetic relationships. Even with a modest number of taxa (eg biological species or weaving traditions) the number of permutations of tree topologies that can be generated is very large, so methods have been developed to detect and sample the most likely trees. In recent years, the most widely used method has been Bayesian Monte Carlo Markov Chain (Bayesian-MCMC) inference [31]. The older method of Maximum Parsimony coupled with bootstrap analysis [32] has also been used for a large number of studies. Both methods aim to find the subset of trees that best fits the available data.

The weaving traditions of SEA are candidates for study by these methods because of their intrinsic cultural significance and because, as noted, their skills and motifs are transferred intergenerationally. Though no previous studies have been done on SEA weaving phylogenies, there is a considerable amount of material available for comparison from linguistic, archaeological and textile scholarship, linked to the continuing interest in this region as a model for cultural dispersal and the development of languages. Aside from the linguistic studies mentioned above there are relevant archaeological studies [33], genetic studies [34]–[36] and analyses of bark-cloth making technologies (discussed below).

In recent years a consensus has emerged about the population history of SEA, despite the challenges involved in collating data from widely differing fields, including genetic studies (Y-chromosome, mitochondrial DNA and whole genome), linguistic studies and archaeological reports. Briefly, the consensus view is that the first modern human peoples in the region arrived before 50,000 BP [37], following a coastal migration route from regions to the west. These migrants were able to colonize much of ISEA via the Sundaland landmass, as a result of lower sea levels during the past ice age (43,000 BP to 12,000 BP approximately). This included the areas that are now the Malay peninsula, Sumatra, Java and Borneo. They pursued a largely pre-neolithic hunter-gatherer lifestyle and occupied inland and coastal regions of much of SEA at low population density. They reached the larger islands in ISEA, including Sulawesi and most of the Indonesian archipelago as far as New Guinea, but not the more distant Pacific islands. The technological achievements of these peoples and subsequent paleolithic migrants are not well understood, but it does appear that they eventually developed farming technology in some places, notably in upland areas of New Guinea, independently of mainland developments.

Around 6000 BP, or perhaps somewhat earlier, a new group of Austronesian-speaking neolithic settlers entered the ISEA region from the Asian mainland, most probably via what is now Taiwan. Genetic studies indicate that they interbred with existing populations to varying degrees, but in many cases retained their Austronesian languages and cultures. They brought with them a neolithic skill-set that eventually enabled them to settle the region at higher densities than the hunter-gatherers did, and they formed settlements that were concentrated in coastal areas. This view is based in large part on linguistic studies, which have demonstrated the connections between Austronesian languages in spite of the (often vast) distances separating them. These studies point to Taiwan as the most recent homeland for this language group, on the basis of a group of highly divergent Austronesian languages that are still found on this island [33]. This is corroborated by archaeological evidence which shows the progressive spread of neolithic technologies from the Asian mainland to Taiwan, subsequently through the Philippines [38] and Borneo, then on to most parts of Indonesia and finally to the furthermost islands of the Pacific. Dissenting views of the history of SEA have also been put forward, identifying Sundaland as a possible point of origin of Austronesian peoples and their languages [39]–[41], though this hypothesis has received less support in recent years.

As mentioned, Austronesian migrants into ISEA brought with them a distinctive set of neolithic technologies. This included farming technologies, ocean-going sailing vessels and navigational skills, stone tools for bark cloth manufacture (a non-woven textile made by beating the inner bark stripped from a tree), and woven cloth. The evidence for bark-cloth manufacture rests mainly on the occurrence of smooth and well-formed stone beaters in archaeological sites on the Asian mainland and similar beaters in ISEA sites. These seem to have been introduced as part of a wider neolithic repertoire of stone-tool making, and their appearance does not necessarily imply that bark-cloth making (eg with wooden beaters) was absent in the region before their arrival. The evidence for the introduction of weaving rests on clay spindle whorls found in Taiwan and the Philippines, on Blust’s identification of weaving-related terms in proto-Austronesian languages [20], and on the present-day distribution of weaving in ISEA. The distribution of the warp ikat technique in particular shows a striking association with speakers of Austronesian languages. Few non-Austronesian speakers weave, and those that do tend to be groups living in close proximity to Austronesian speakers.

Bark-cloth making had a wide distribution throughout mainland SEA and ISEA until recent historical times, and is of particular importance amongst Austronesian speakers in Oceania. Tolstoy [42] and Larsen [43] studied distribution and technologies of bark-cloth in Oceania using phylogenetic methods. Their work shows that the current distribution of bark cloth can be explained in part by tree-like evolutionary processes, but that contacts (and horizontal transfer of ideas) seem to have persisted even between remote islands for some time after initial colonization. The geographic distribution of bark-cloth in the ISEA region as a whole is more complex than that of warp ikat however, and includes both Austronesian and non-Austronesian language speakers.

### The Warp Ikat Technique

**Figure 1:**
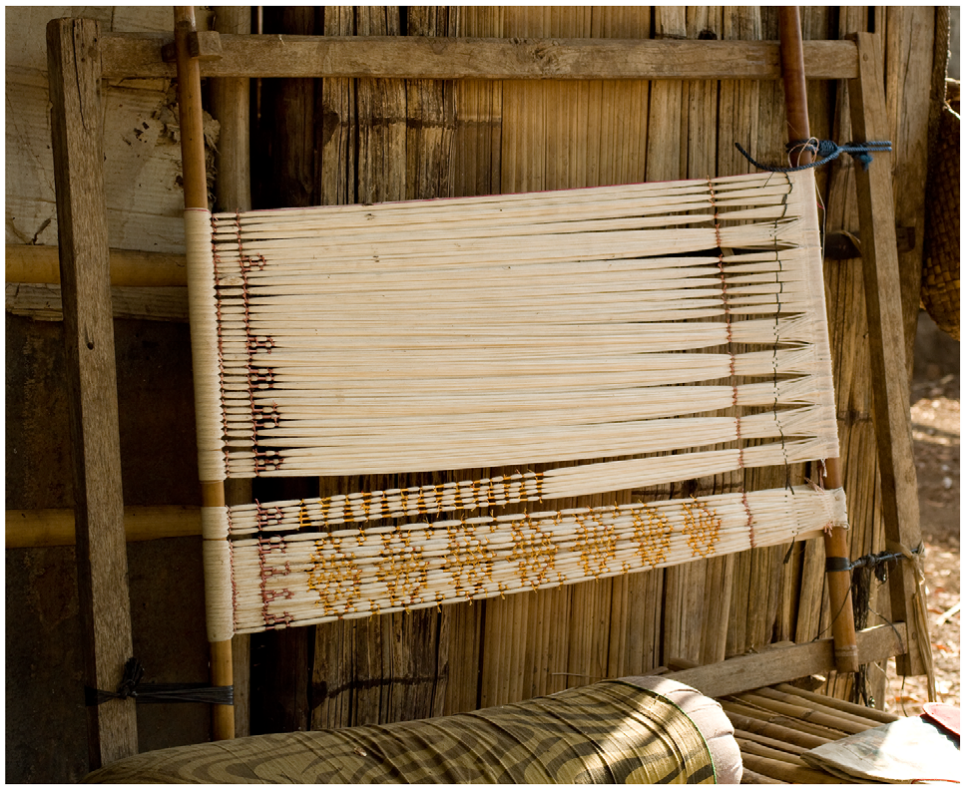
The warp ikat process: making the resists. Warp threads stretched on a frame, with partly tied ikat resists, on the island of Alor. Weavers use essentially the same method, using a similar frame, in all the areas considered in this study.

Woven textiles are defined as those created by interweaving flexible threads (warp and weft) at right angles. For convenience, this is usually done with the warp threads stretched between two parallel beams, called a loom. The defining characteristic of warp ikat is that the warp foundation of the textile is decorated with dyed designs before weaving. This is done by attaching the warp to a frame and then tying strips of resist material tightly around bundles of a few warp threads at a time (Fig.1). The tied warp is then dyed and the resists are removed, leaving a design in white against a colored ground. In some cases, several tying and dyeing steps are used in sequence to make designs with several colors.

**Figure 2:**
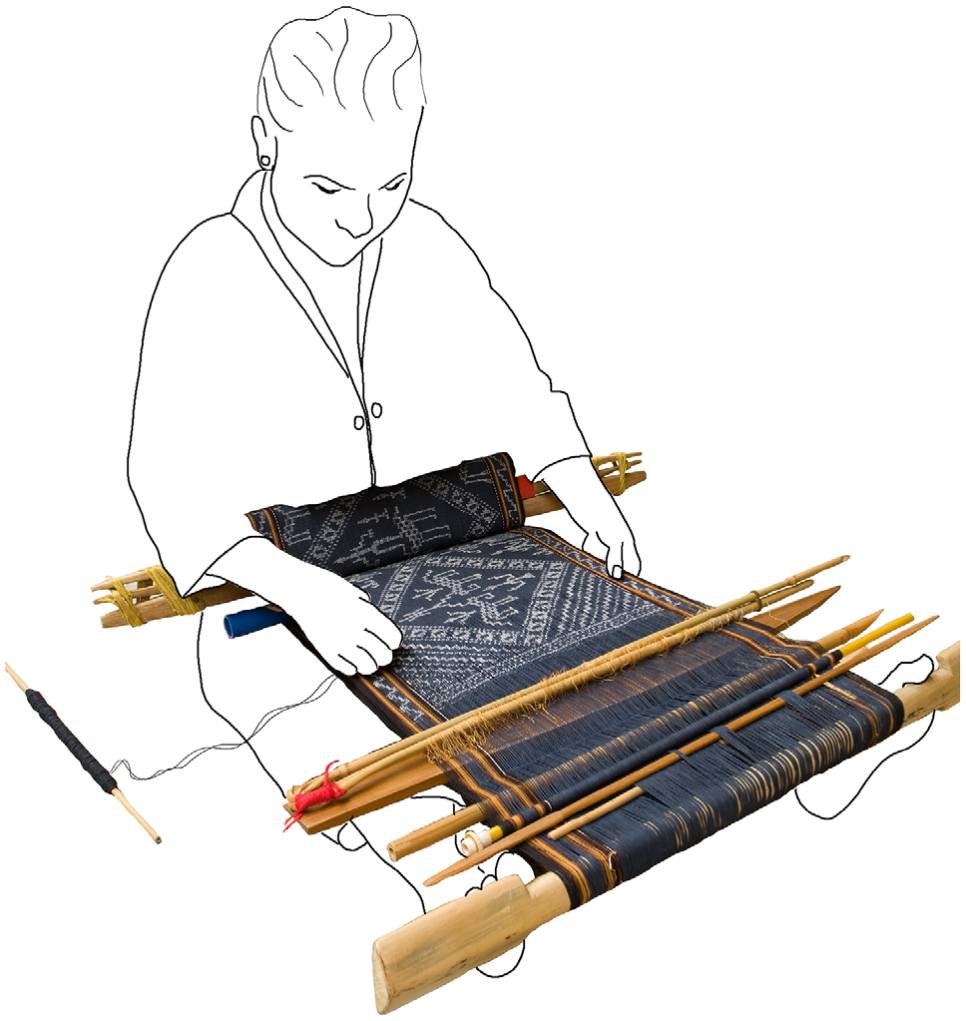
The warp ikat process: weaving. Sketch showing how a backstrap loom is used by weavers on Hainan Island to weave ikat cloth. Weavers use similar backstrap looms in all the areas considered in this study, though in most areas outside of Hainan weavers attach the warp beam to a fixed point rather than bracing it directly with the feet.

**Figure 3:**
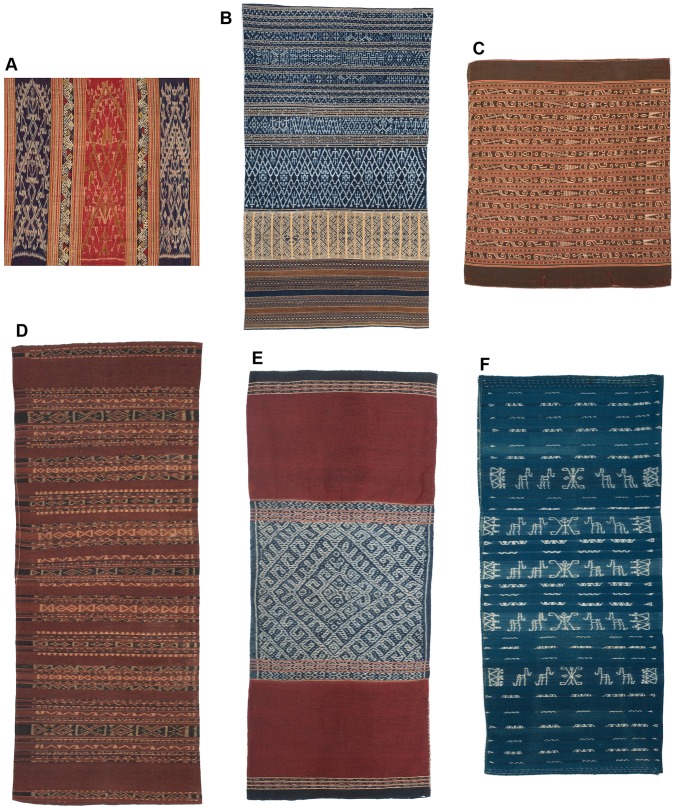
Examples of Southeast Asian textiles with ikat decoration. A: detail of Tai weft ikat cloth with bands of supplementary weft decoration, formerly part of a tubeskirt with a waist band above and a narrow band of plain cloth below, B: Meifu Li tubeskirt from Hainan Island, made up of 5 panels of warp ikat decorated cloth and one panel of supplementary weft decoration (lighter color), C: Iban tubeskirt from Borneo with warp ikat and supplementary warp decoration, single panel. D: Lamaholot tubeskirt from Ili Api (eastern Flores) made up of two panels of warp ikat and warp stripes, E: Timor tubeskirt from Belu regency, made up of 3 panels with warp ikat and warp stripes, F: tubeskirt made by Ngada people from central Flores, made up of three panels of warp ikat decorated fabric.

The dyed and patterned warp is then mounted on a simple loom consisting of a breast beam (nearest to the weaver) and a warp beam (furthest from the weaver). The loom is tensioned by means of a strap that is attached to the breast beam and passes behind the weaver’s back. In Hainan Island and a few areas of the Asian mainland the warp beam is braced directly by the weaver’s feet (Fig.2), however in most other regions of SEA the warp beam is tied to a fixed point such as a tree or a couple of stakes in the ground. The sequence of dyeing followed by weaving gives warp ikat its characteristic appearance and soft outlines (Fig.3).

Aside from the basic tying and dyeing process, several aspects are shared by all the weaving traditions considered in this study:

1. Weavers use a simple body-tensioned loom as mentioned, as opposed to the more complex frame looms that are also present in most parts of Asia.
2. The warp is circular and continuous, and the loom is used without a reed (a device used on more sophisticated looms to control the spacing of warp threads).
3. The ikat designs are usually interspersed with plain stripes in the warp direction, or narrow bands of supplementary warp patterning.
4. Most textiles include bands patterned with simple ikat dashes, in addition to wider bands with more complex motifs.

In this study my aims are to answer some of the questions that were raised in the Introduction, specifically:

1. Do the ikat weaving traditions of Southeast Asia share a common origin?
2. Given that weaving techniques and motifs are passed from generation to generation, can a phylogenetic tree and taxonomy of ikat weaving traditions be reconstructed from their present-day characteristics?
3. Assuming that there was a single ancestral tradition of SEA warp ikat weaving, what were its characteristics?
4. Is the theory of the origin of Asian weaving motifs in the Dong-Son culture correct?

Answers are provided to these questions by means of a dataset consisting of decorative characters of the weaving traditions, analyzed using phylogenetic methods.

## Materials and Methods

### 1) Taxa

**Figure 4:**
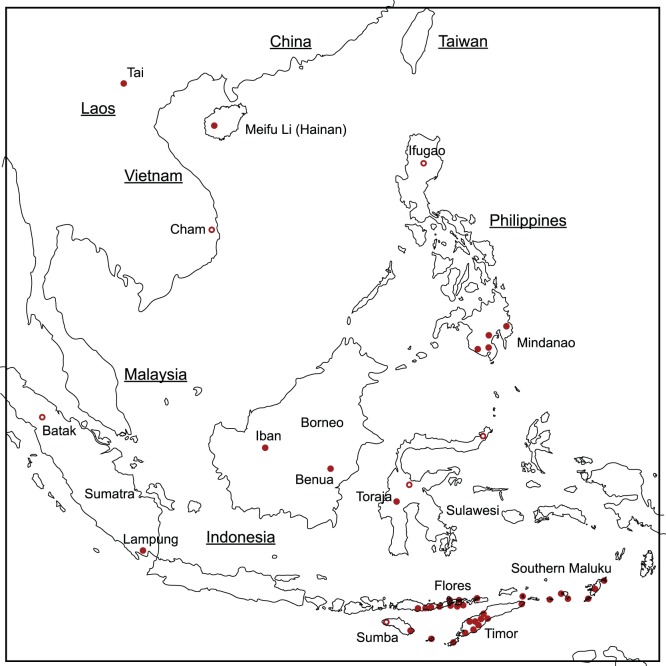
Locations of warp ikat-weaving traditions (taxa). Traditions included in this study are shown with solid red circles, warp-ikat traditions that were not included are shown as hollow circles.

The taxa included in this study are 36 weaving traditions. The criteria for inclusion of taxa are firstly that their textiles should be distinct, recognizable and well defined in the existing literature. Secondly, sufficient examples are available for study (in most cases 30 or more textiles). Thirdly, sufficient characters can be identified to ensure that the taxa are informative from a phylogenetic standpoint. The weaving traditions correspond to broadly recognized cultural groups with distinct languages or dialects, in most cases with self-recognized identities. They are drawn from a wide area of SEA, including the Asian mainland (Vietnam and Laos), China (Hainan Island), the southern Philippines (Mindanao) and the Indonesian archipelago. The locations of weaving taxa are shown in Fig.4 and a list of definitions is given in TaxaChars S1. Weaving groups are defined as narrowly as the data available allows. In the case of the Flores and Lamaholot weaving traditions (for example) published data allows textiles from neighboring groups to be distinguished. In the case of the Mindanao group (comprising Bagobo, T’boli, B’laan, Mandaya and Kulaman cultures) published information on textiles that I was able to obtain was not large enough or well characterized enough to distinguish these related traditions, so they are grouped together in this study as a single taxon. In the case of the complex (and sometimes overlapping) traditions of Flores and of Timor I have followed the definitions of weaving traditions of Hamilton et al [8] and Yeager and Jacobson [44] respectively.

Photographic images and examples of textiles were used as the main data source: these comprised 46 print publications [1]–[12], [44]–[77] and three online museum collections [78]–[80], yielding approximately 2100 usable examples of textiles. These were supplemented with 170 textiles that I collected and/or photographed in the field. In order to obtain background information and to assess the completeness of the data I made field trips to several of the weaving groups in this study during 2009–2011, visiting Meifu Li weavers on Hainan Island, Lio and Sikka weavers in central Flores, Lamalera and Ili Api weavers in the Lamaholot-speaking areas of eastern Flores, Amarasi, Amanuban, Biboki and Belu weavers in Timor (2 separate visits), Toraja weavers in Sulawesi, Iban weavers in Kalimantan and Borneo. A group of 50 textiles that I collected from the Meifu Li weaving tradition on Hainan Island is particularly important in this respect since this tradition is not well documented and there are only an handful of published examples. The Meifu Li are one of the very few non-Austronesian groups in SEA with a rich tradition of warp ikat textiles.

Though the focus of this work is warp ikat, a group of weft ikat textiles from Tai Daeng, Tai Dam, Tai Mai Chau and related weavers living in the regions near Muang Phan and Xam Nuea (border region between northern Vietnam and Laos) are included as a single taxon. These textiles form a distinctive group that appears to have absorbed little Indian influence on motifs and design layout, in contrast to most Tai weft-ikat traditions in SEA. Instead, they seem to represent a transposition of older warp-ikat motifs to the weft ikat format [7], [60], preserving the division into narrow bands and some of the motifs that characterize indigenous SEA warp ikat. This group of weavings seems more distantly related to the warp ikat of the other taxa and is used as the outgroup in this study for the purposes of rooting the phylogenetic trees.

A few warp ikat traditions were excluded from the study because there are insufficient documented examples of textiles available: these included the warp ikat made in the northern Philippines (Luzon) and the eastern regions of Sulawesi (Poso and Bentenan). The Batak tradition from northern Sumatra, which has been comprehensively documented by Niessen [10], was also excluded because the cloths from this tradition have a different layout and motifs compared with the other taxa in this study and may have a different origin. Batak cloths are characterized by a small number of simple motifs used in a repeating fashion over a relatively large area of cloth, and by the presence of motifs such as the chevron that are absent in warp ikat from other areas.

In addition to the SEA textiles, I surveyed a group of 111 Indian trade textiles, mainly from the 17^th^–19^th^ centuries, in order to identify characters that may be derived from this source and to exclude them from the phylogenetic study [81], [82]. Bronze drums the Indonesian National Museum in Jakarta that have been found in the Indonesian archipelago were surveyed, along with published photographs of Dong Son artifacts [83], in order to develop a list of Dong Son geometric motifs. These were not included in the phylogenetic research, but were used for comparison with ancestral states for the ikat weaving traditions (see Discussion section).

### 2) Characters

The ‘characters’ included in a phylogenetic study are definable characteristics shared by the taxa that can be coded as present or absent. In this case the characters are decorative features, the majority of which are motifs. Dyes and fiber types were not included since these are expected to be dependent to some degree on materials that are available in local environments, especially given the wide geographic area spanned by this study.

A classification system was developed specifically for recognizing and coding geometric warp ikat motifs. This classifies geometric motifs according to the primitives of which they are composed. Primitives are defined as simple shapes that ikat weavers use to construct many of their designs. This system helps with identifying motifs that are fundamentally similar, despite differences in rendering across textiles and weaving traditions. Weavers may change the arrangement or scale of motifs, stretching or expanding them, but it is comparatively rare for a weaver to create a new motif, and rarer still for a weaver to spontaneously develop or adopt a new primitive.

**Figure 5:**
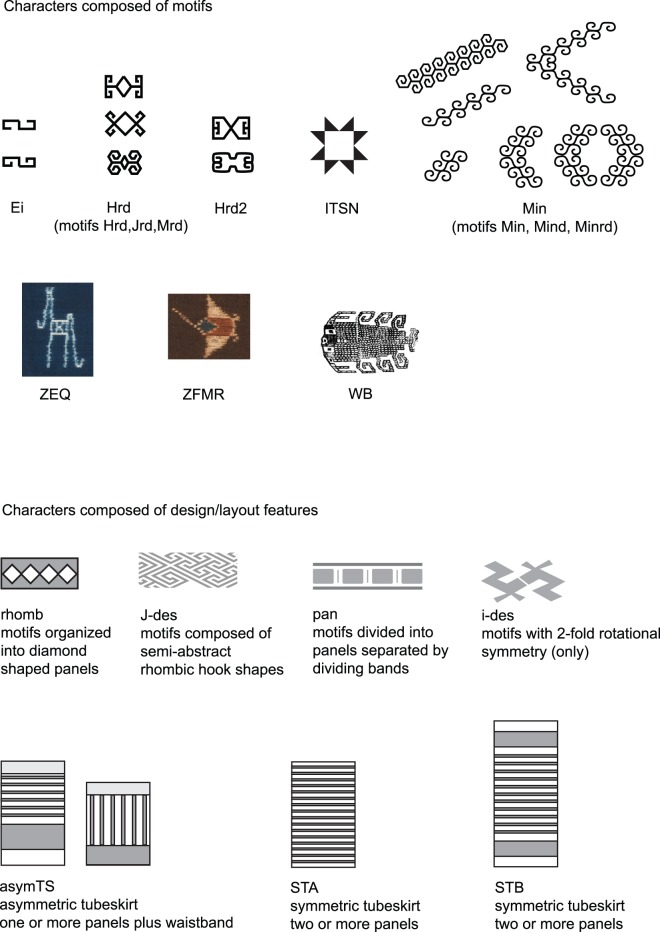
Examples of characters and their associated decorative features.

In some cases the characters in this study correspond to individual motifs, in other cases characters correspond to a small group of similar motifs that are equivalent from a weaver’s perspective. For example Jnrd and Mnrd, the square and hexagonal/rounded forms of hook-and-rhomb motifs, are grouped together as one character since weavers generally regard them as being the same. Equivalent motifs can be detected in practice by identifying groups of motifs that are present on similar textiles and where variations seem arbitrary, or related to the skill of the weaver in laying out the design. Grouping such variants where the weaver’s intent is the same into a single character is important to avoid double-counting. A further 11 characters based on design features and textile types were included in the study. These characters are based on broader design features, such as the arrangement of decorative bands or the use of certain kinds of symmetry and repetitive features in textiles. The complete list of more than 100 characters and motifs is given in TaxaChars S1 and Motifs S1, and examples of characters are illustrated in Fig.5.

Characters that were phylogenetically uninformative because they were present in all of the taxa or less than two taxa were excluded from the dataset. Several motifs that were apparently copied from European textiles were also excluded, as well as a further 18 characters that may derived from Indian trade textiles (patola and other types). The relationship between Indian trade textiles and Indonesian textiles is a complex one: in some cases the Indian examples may be based on Indonesian prototypes or vice versa. However, since these relationships cannot be untangled easily the entire set of characters that belongs to both sets of textiles was excluded from the study. This left a residual set of 110 characters that were presumed to be indigenous. The result of this survey and coding work is a table of taxa and characters coded according to their presence (1) or absence (0) in each taxon (Data S1). This was converted to file formats usable by phylogenetic software using Mesquite [84], which was also used to visualize phylogenetic trees and to deduce ancestral states.

### 3) Analysis

**Figure 6:**
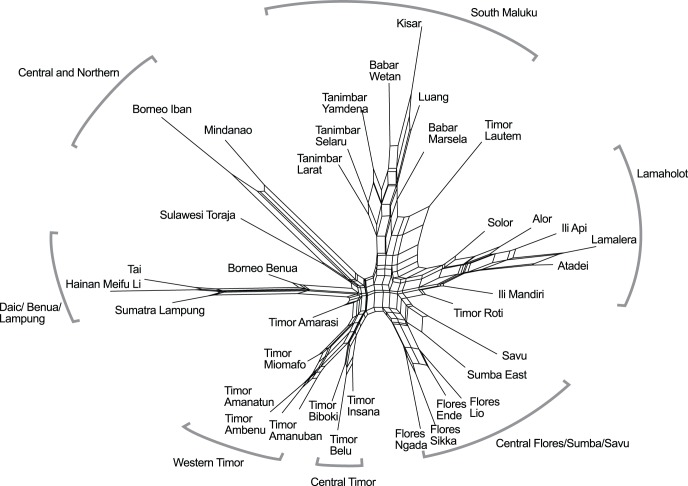
Neighbornet plot of weaving taxa. Clusters, which mostly correspond to geographic regions, are labeled at the edges.

As a first step, a Neighbornet plot was prepared using SplitsTree [85]. This model-independent plot provides a means of visualizing relationships in the dataset. Neighbornet plots can be helpful for determining to what degree a dataset is tree-like (ie contains a phylogenetic signal) as opposed to net-like. They can also help to show what natural groupings exist between taxa. Delta scores [86], [87] were also calculated for the dataset as a whole and for individual taxa, using SplitsTree. This measure gives an indication of the amount of non tree-like signal present on a taxon-by-taxon basis. The Neighbornet plot is shown in Fig.6 and the delta scores are listed in Table 1.

**Table 1:** Delta Scores for individual taxa, ordered from lowest to highest.

Taxon	Delta Score
Lamalera	0.31
Timor Amanatun	0.32
Babar Marsela	0.32
Timor Ambenu	0.32
Atadei	0.32
Hainan Meifu Li	0.33
Sumatra Lampung	0.33
Timor Amanuban	0.33
Ili Api	0.33
Luang	0.33
Alor	0.33
Tai	0.34
Kisar	0.34
Tanimbar Larat	0.34
Tanimbar Yamdena	0.34
Timor Biboki	0.34
Babar Wetan	0.34
Timor Insana	0.35
Tanimbar Selaru	0.35
Borneo Benua	0.35
Timor Miomafo	0.36
Timor Roti	0.36
Ili Mandiri	0.36
Flores Ngada	0.36
Flores Ende	0.36
Mindanao	0.36
Solor	0.36
Flores Sikka	0.36
Borneo Iban	0.37
Sulawesi Toraja	0.37
Timor Malaka	0.38
Flores Lio	0.38
Timor Amarasi	0.38
Savu	0.38
Timor Lautem	0.39
Sumba East	0.41

Delta scores are a measure of the amount of reticulate (non tree-like) signal in the data.

**Figure 7:**
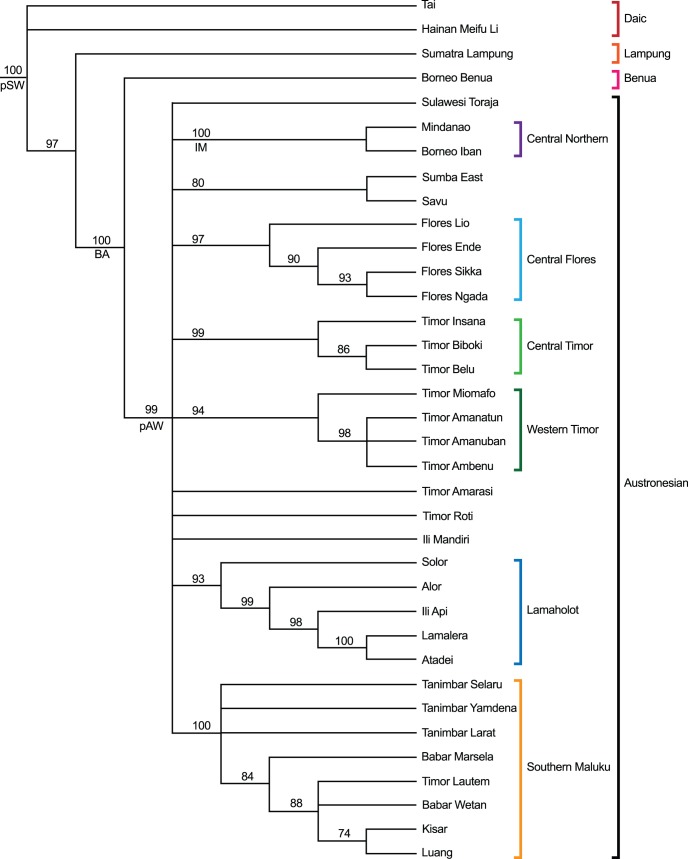
Results of Bayesian analysis of weaving taxa. 70% majority rule cladogram of 1000 trees sampled from a 10,000,000-step MCMC search using flat priors, variable character-change rate. Clade frequencies are shown as percentages behind each node. Proposed taxonomy of weaving traditions shown by the bracketed taxa on the right hand side, based on clades with support of 90% or better.

The phylogenetic aspect of the data was investigated by searching for best-fit phylogenetic trees via Bayesian Markov Chain Monte Carlo analysis, using MrBayes [31]. The Bayesian-MCMC approach identifies the region of treespace that contains the most probable trees (ie those that best reproduce the observed data) using a Markov Chain search, and samples this region selectively. The result is a sample of most probable trees that can be combined to produce a consensus view of the best-fit tree. For the Bayesian analysis two slightly different models were tested: the first was the simplest possible model with ‘flat’ prior assumptions, using data coded as ‘standard’ following the method of Matthews et al [29] and an equal rate of change assumed for all characters. The second model modified this simple model by allowing characters to change at different rates, using a gamma distribution modeled with four steps. The Bayesian MCMC procedure was run for both models using standard methods: four MCMC chains, three ‘hot’ and one ‘cold’, with two analyses run in parallel to assess convergence and a total of 10 million chain steps, sampled every 1000 steps. The first 2.5 million steps were discarded (burn-in) to ensure that the MCMC samples were taken from the maximum-probability region of tree space. Convergence figures during the MrBayes runs indicated that this region was in fact found much sooner, after around 300,000–500,000 steps. The resulting samples of 1000 best-fit trees were summarized by calculating the 70% majority rule consensus tree using Mesquite. This means that the final consensus tree displays only clades that appear in 70% or more of the sample of best-fit trees. The consensus tree for the second model is shown in Fig.7. The Retention Index (RI) was also calculated in Mesquite for an indication of the goodness of fit to the dataset as a whole [88]. Bayes factors [89] were computed for each model using MrBayes. Ancestral states were also deduced for several nodes in the Bayesian tree using the Likelihood method in Mesquite. These nodes are marked pAW, pSW, IM and BA in Fig.7. The sets of ancestral characters for the nodes at pAW and pSW are given in Table 2.

**Table 2:** Ancestral States for the mainland root weaving tradition (pSW) and the Austronesian root weaving tradition (pAW).

Node	>95% likelihood	80–95% likelihood
pSW (proto-Southeast Asian warp ikat tradition)	A2, C1, DB, Efr, Er, J, Jnrd, Jnrd-IN, J-des, rhomb,pan, i-des, asymTS, CBR	ZHhead
pAW (proto Austronesian warp ikat tradition)	BT, C1, DB, DBT1, DBT, Hrd, Jnrd, STB, SBL	KrW-dots, KrW, Hrd-ext

Ancestral states are identified by likelihood analysis based on the 70% consensus tree from Bayesian analysis (Mk1 model, symmetric reversible state changes).

## Results and Comparison of Models

The Neighbornet plot (Fig.6) has a starfish-like shape, with weaving traditions mostly falling into clusters along the ‘arms’, indicating that the data contains resolvable structure. The weaving taxa cluster into groups that correspond (roughly speaking) to geographic regions: these are shown by the curved brackets around the plot. The delta score for the entire dataset is 0.35 (versus a theoretical range from 0 to 1, in order of increasing net-like character), and some net-like structure is apparent at the center of the plot. Taken together, these show that the relationships between taxa are not purely tree-like.

Delta scores for the individual taxa range between 0.31 and 0.41. The weaving traditions of the islands of Sumba, Timor Lautem and Savu have the highest scores, and these are also the taxa that tend to lie in the regions between the clusters of taxa on the Neighbornet plot. The relatively high delta score for the Sumba taxon is interesting: this prominent ikat weaving tradition has been active in exporting cloths to neighboring regions, and is noted for sometimes producing copies of the designs of surrounding regions for export purposes. In this respect the Sumba weaving tradition is unusual: most ikat weavers in SEA weave for their own use or for sale in a very localized area. The taxon with the lowest delta score is that of Lamalera. The conservatism of this tradition, particularly for ceremonial textiles, has already been mentioned in the Introduction to this study.

**Figure 8:**
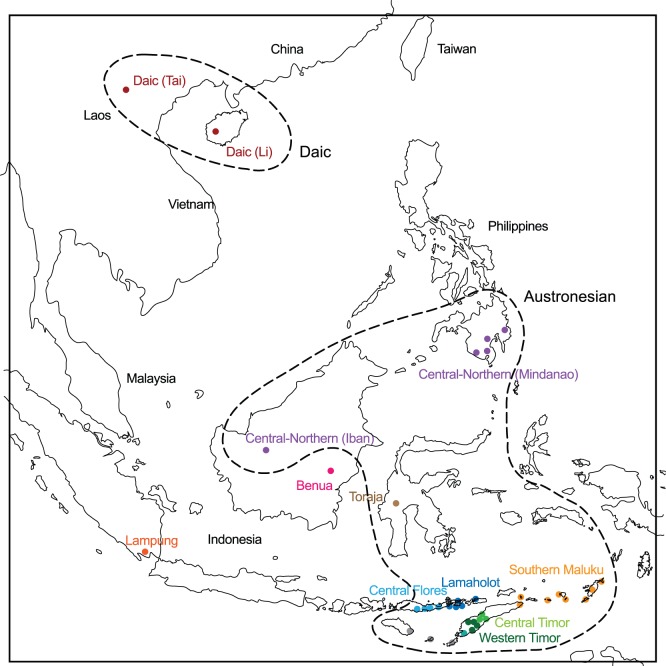
Geographic distribution of warp ikat weaving clades and sub-clades. Weaving clades correspond to those labeled in the Bayesian tree in Fig.7. The Daic and Austronesian weaving clades are indicated by dotted lines. The clades have some similarities (and some differences) with groupings identified in linguistic studies.

**Figure 9:**
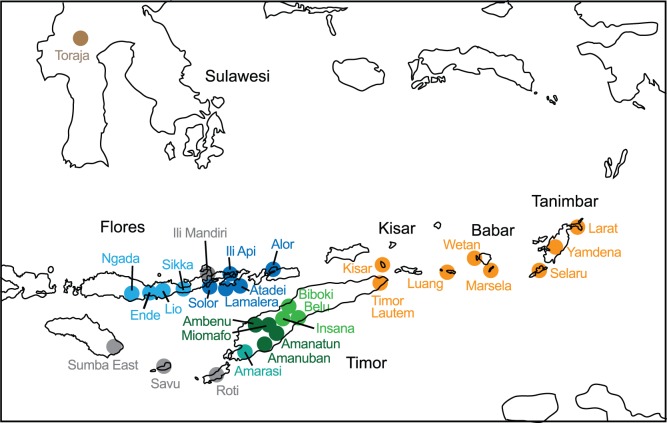
Enlargement of the southeastern corner of **Fig.8**. Weaving traditions with uncertain or blended affiliation are shown in grey.

In the Bayesian analysis both models yielded consensus trees with a similar topology. The Bayes factor between these two models is 35 log units in favor of the variable rate model, indicating that it represents the data significantly better than the fixed rate model. The variable rate model also produced a better-resolved tree, with support values for most of the major clades of 90% or above, so this is the one that is presented here. The clades in this tree, which I will call the Bayesian tree, reflect similar groupings to those identified by the Neighbornet plot. The best-supported clades are used to define a taxonomy for SEA weaving traditions, which are indicated by the bracketed taxa on the right hand side of Fig.7. The corresponding geographic locations of the taxa and clades are shown in Figs.8 and 9.

In broad terms the Bayesian tree groups most of the Austronesian-language speaking weaving traditions in a single clade with several sub-clades. The weaving traditions of the Daic groups (Tai and Meifu Li), the Lampung people, and Benua people are placed in 3 separate clades. Ancestral states deduced at the basal node pSW and the base of the Austronesian clade pAW, yielded 12 and 15 ancestral characters at pSW and pAW respectively, most of which have likelihoods of 95% or better (Table 2). It is very unlikely that warp ikat with these complex sets of characters arose independently and by chance in two or more parts of SEA, so the results confirm that the SEA warp ikat weaving traditions do indeed form a single group with common ancestry.

### Tree-ness and Confidence Limits

The Retention Index (RI) for the Bayesian tree is 0.5. This value lies within the range of published RI values for datasets that are claimed to contain a phylogenetic signal [27]. It confirms the picture given by the delta scores that the relationships between taxa include both tree-like and net-like components. Non tree-like signal may come from several sources: horizontal copying of motifs between neighboring traditions, conflicting tree-like signals, or ‘noise’ in the data due to inaccurate coding. Most probably, a mixture of factors is present. The presence of some horizontal exchange of motifs is virtually certain, given that numerous examples of this (eg copying from Indian and European textiles) were encountered and excluded from the data during the coding process. It is likely that undetected examples of copying within the study region remain.

Given the partly phylogenetic character of the data, the relevant question is to what extent the Bayesian tree accurately represents the phylogenetic component, and hence the line of descent of the weaving traditions. The reasons for thinking that it is both the best available and a reasonably good reflection of the phylogenetic signal are firstly the high degree of agreement amongst the trees in best-fit Bayesian sample, yielding support values in the range of 94–100 for most of the major clades. Secondly, the close correspondence between the major clades identified in the Neighbornet and Bayesian analyses. Thirdly, the clades identified correspond well with existing understanding of the relationships between SEA weaving traditions. In this paper I therefore put forward the Bayesian tree and the associated taxonomy as the ‘current best model’ of the phylogenetic descent of SEA ikat weaving traditions.

As mentioned, many of the groupings defined in the Neighbornet plot and by the Bayesian analysis correspond to groupings used implicitly or explicitly by textile scholars. The central Flores group and the Lamaholot group were recognized as distinct by Barnes [4] and by Hamilton and his co-authors [8]. The Southern Maluku group has been identified and described in a recent essay by van Dijk [90], who made a distinction between the islands at the western end of this region (Kisar, Luang and Babar) and the Tanimbar group, a division that is also reflected in the Bayesian tree. The Daic group has been identified and discussed by Gittinger and Lefferts [7] and by Howard [60]. Some other groupings and divisions require more interpretation to understand them fully.

It is noteworthy that the best-supported divisions (clades) are those between groups of weaving traditions that are further apart geographically, in other words between traditions where horizontal transfer is less likely. Conversely, there are several unresolved parts of the Bayesian consensus tree (polytomies) between neighboring weaving traditions, such as the Timor Amanuban, Amanatun and Ambenu traditions and the three Tanimbar traditions, suggesting that other processes, of which copying of neighboring motifs is a likely candidate, have played a significant role in these localized regions. The relationships between traditions are correspondingly less tree-like at a local level, at least in some areas.

## Discussion

The Bayesian tree and ancestral state analysis are a rich source of information about the evolution of weaving traditions in particular, and Southeast Asian cultures in general. I will firstly consider the implications of the tree for the origins of ikat weaving in SEA, and how these compare with the broader understanding of the origins of SEA cultures. I will then look in more detail at the characteristics of the ancestral weaving tradition, and use these to examine the Dong-Son hypothesis. Finally, I will consider some of the fine-grained information present in the tree and its implications for understanding the relationships between, and distribution of, weaving traditions that exist today.

### 1. Overview and Interpretation of the Phylogenetic Tree

As well as providing a taxonomy of ikat weaving, the phylogenetic tree is a reconstruction of the history and descent of this tradition. A bifurcation (node) in the tree indicates a common ancestor of two or more traditions that existed at some point in the past. Polytomies (nodes with more than one branch) indicate unresolved branching structure. As noted, the standout feature of the tree is that it groups all but two of the Austronesian speaking weaving traditions into a single clade with multiple sub-clades and a polytomy at the base. This feature may be the result of the rapid evolution of Austronesian group and its sub-clades (relative to the entire tree), and/or a lack of sufficient data to resolve finer detail.

As noted, the phylogenetic tree shows that the Austronesian weaving traditions share a common ancestor with Daic ikat weaving traditions on the Asian mainland and on Hainan Island. The simplest interpretation of this is that ikat weaving originated on the Asian mainland and subsequently spread to ISEA through several dispersal events. The major dispersal is that represented by the Austronesian clade. Separate dispersal events brought ikat weaving to Hainan Island, the Sumatran peninsula, and possibly to Borneo as well (Benua tradition). This interpretation accords well with the wider context of weaving in the region, since many of the motifs of warp ikat weaving that I have identified as ancestral to the tradition are also present in other (non-ikat) weaving traditions on the Asian mainland.

Are any other interpretations of the phylogenetic tree, beside a mainland origin for warp ikat weaving, tenable? Might ikat weaving have originated ‘offshore’ somewhere in ISEA (say, Borneo or the Philippines) and then spread to the Asian mainland? This seems unlikely, since a convoluted geographic route would be required get the Tai and Meifu Li weaving traditions (for example) from an offshore origin to their current locations in a way that is consistent with the phylogenetic linkages. Furthermore, there is no evidence (that I am aware of) for any material culture or language that has appeared first in ISEA in pre-historic times and subsequently spread to in the Asian mainland, though there are plenty of examples in the other direction. There is also no evidence of prehistoric migration from ISEA to the Asian mainland, though some later and localized examples exist, notably the migrations of Chamic speakers [91].

### 2. Comparison with Austronesian Language Phylogeny

As mentioned, the phylogenetic tree for Austronesian languages shares some broadly similar features with the ikat weaving tree, in particular the radiation of a similar geographic group of Austronesian languages in ISEA from a common origin. The linguistic tree has been interpreted as reflecting the migratory history and descent of the Austronesian-speaking peoples, consisting of movement from the Asian mainland to Taiwan followed by a pause, then a relatively rapid migration from Taiwan across a wide area of ISEA and the Pacific [33]. It is tempting therefore to identify the radiation of weaving groups at the base of the Austronesian clade with this migration event into ISEA by Austronesian settlers. This explanation, that Austronesian migrants brought the warp ikat technique with them during their migrations has the advantage of simplicity, and readily accounts for the geographic distribution of warp ikat that is seen today. The phylogenetic tree for ikat weaving includes no absolute dates however, so a later dispersal of the ikat weaving technique, subsequent to Austronesian migrations, cannot be ruled out by phylogenetic analysis alone.

Support for the view that Austronesian settlers brought weaving (but not necessarily ikat) with them during their migration from the Asian mainland comes from reconstruction of the proto-Austronesian (PAN) and proto-Malayo-Polynesian (PMP) languages of these early settlers by Blust [20]. His reconstructions indicate that both PAN and PMP speakers had vocabulary related to weaving, including words for the breast beam of a loom, the weaver’s sword used for beating-in the weft and a spindle used in making spun yarn. In addition to this, they had words for the long cloths used for carrying children on the back or the hip, and for mat making and cordage. Blust was also able to identify a PMP word for tubeskirt (tapis).

Ikat weaving is absent in Taiwan amongst aboriginal Austronesian speakers, despite the identification of this region as a point of origin or key staging post for the Austronesian diaspora. It is present however immediately to the north of Taiwan in the Ryuku Islands and immediately to the south in the northern Philippines. Patchy distributions are a characteristic feature of technologies such as ikat weaving, especially amongst small populations. Many Austronesian-speaking groups do not make ikat and many do not weave at all, so absence in a given location is not especially remarkable. Weaving with a backstrap loom is practiced in Taiwan by Austronesian-speaking groups however, and the loom used is a foot-braced kind similar to that used by Li weavers on Hainan and by some weavers on the Asian mainland. Whether the warp ikat technique was lost at some point from Taiwan, perhaps under the influence of later developments from mainland China, or did not take hold there is not known.

As noted, there are two Austronesian-speaking ikat weaving groups that are not part of the main Austronesian clade: these are the Lampung weavers of Sumatra and the Benua weavers of Borneo. The phylogenetic tree suggests that these groups split off from the ancestral tradition at an earlier date. In the case of Lampung weaving, given its high divergence from the main Austronesian group and the fact that the phylogenetic tree places it closer to mainland (Daic) traditions, it is unlikely that it arrived at its current position as a result of the main Austronesian diaspora. Gittinger and Lefferts [7], who compared the weaving traditions of the Lampung and Daic groups, also noted similarities between these traditions and suggested a direct connection with the Asian mainland. A separate migration route from the Asian mainland must also be considered as a possible explanation for the origin of the Benua weaving tradition of Borneo.

### 3. Reconstructing Ancestral Weaving Traditions

**Figure 10:**
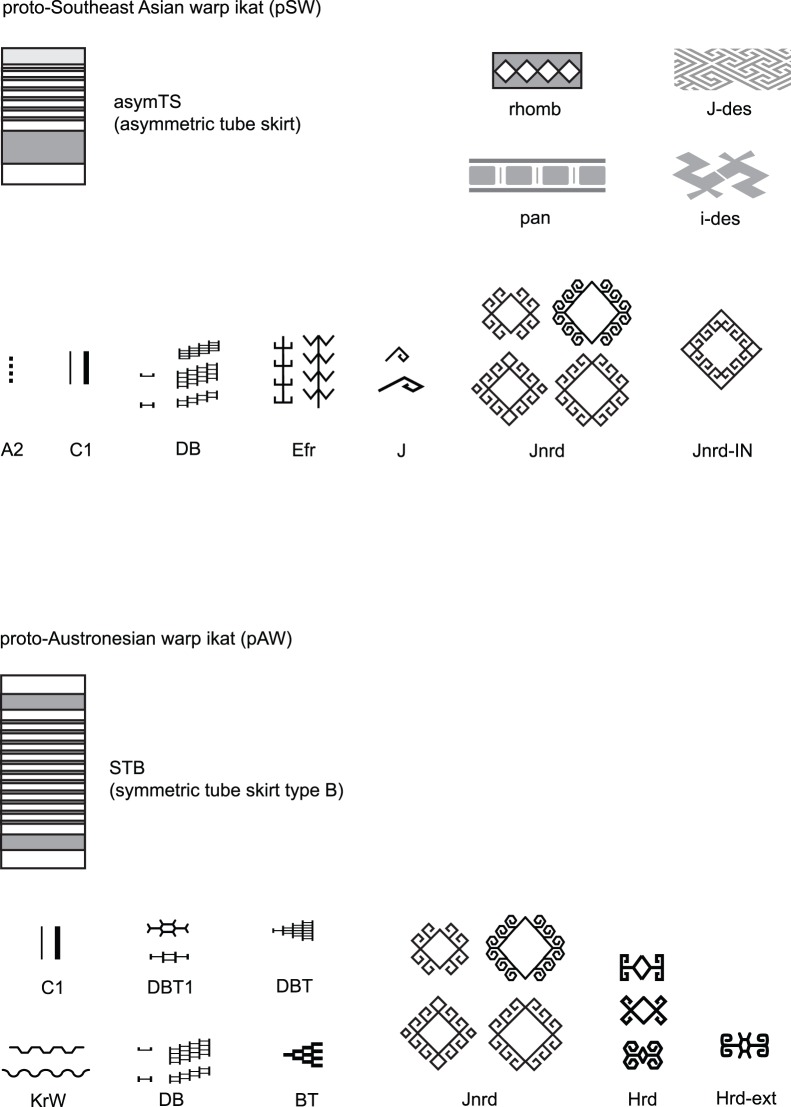
Reconstruction of the characters of the ancestral traditions of SEA warp ikat. The ancestral characters of the pSW tradition, presumed to be located on the Asian mainland, and of the proto-Austronesian (pAW) tradition.

One of the most powerful aspects of phylogenetic analysis is that it allows some of the key features of the ancestral traditions in the phylogenetic tree to be reconstructed. The last common ancestral tradition for all the ikat weaving taxa in this study corresponds to the node pSW (proto-Southeast Asian Warp ikat), which was located on the Asian mainland. The last common ancestral tradition for Austronesian ikat weaving is at the node pAW, which presumably existed at an offshore location, perhaps in the Philippines or Borneo. The ancestral characters for pSW are summarized in Table 2 (Results section). To this list, we can add the 4 characters, mentioned in the Introduction, that are shared by all ikat-weaving traditions in SEA. This gives us the following reconstruction of this tradition: pSW weavers wove warp ikat using a continuous circular warp, mounted on a simple loom tensioned with a backstrap. They used this cloth to make tubeskirts of asymmetric shape (asymTS), composed of several bands of cloth. The decoration on their textiles was sometimes organized around a main band of diamond shapes (rhomb) with subsidiary bands of decoration, and they sometimes divided their ikat motifs up into panels (pan). They also used interlocking band motifs (J-des) and motifs with twofold rotational symmetry (i-des). Specific motifs included hook-and-rhomb motifs (Jnrd/Mnrd and Jnrd-IN), hooks attached to other motifs (J) and abstract plant-like shapes (Efr). They used the dash-and-tick technique to construct many of their motifs (DB), and they finished the ends of their ikat designs with rows of dots or with lines oriented along the weft direction (A2 and C1). These ancestral characters are shown pictorially in Fig.10. Comparing this list with the entire set of characters, it is skewed towards simpler motifs and ‘structural’ features such as constructing motifs from dash-and-tick shapes. These features were highly conserved by successive generations of weavers.

The proto-Austronesian ikat weaving tradition (pAW) is similar to pSW, except that a switch from asymmetric tubeskirts to symmetric types occurred in the interval between these two. pAW also adds some variations on dash-and-tick motifs, including triangles made up of dashes and ticks (DBT, DBT1 and BT) more hook-and-rhomb designs (Hrd and Hrd-ext) and wave-like motifs (KrW). Some of these characters, such as Hrd and its variants, also occur in some other kinds of present day mainland Asian weaving (such as Tai supplementary weft motifs) so these may represent Austronesian preferences rather than innovations. Overall, the motifs of the two root traditions are similar, which is not surprising given their close relationship.

The time depths of these ancestral traditions is a matter of conjecture, but if the identification of warp ikat dispersal with Austronesian migration is correct, pSW must have existed before 6000 BP, while archaeological data relating to the earliest settlements in the north of Philippines [38] would place pAW at around 4000–4500 BP.

Aside from what is present in the ancestral states analysis, it is also interesting to reflect on what is absent. For example, the wide distribution of lizard/crocodile motifs (character ZCR) across ISEA might lead us to suppose that these motifs are ancestral to all SEA weaving. In fact, the statistical analysis on the Bayesian consensus tree shows that the occurrences of these motifs are sporadic with no clear pattern, and that the most likely explanation for the current distribution is that they were invented independently on several occasions by different groups (Hainan, Benua, Central Northern, Central Timor, Western Timor and Lamaholot clades). These compelling motifs seem to have been ‘borrowed from nature’ on multiple occasions. With the benefit of hindsight this is not surprising, given the large and impressive crocodiles and other reptiles that are a constant presence in people’s lives in much of SEA. This also underlines the benefits of a statistical approach, versus attempting to draw conclusions about the ancestry of a tradition based on consideration of one or two prominent motifs.

### 4. Evaluating the Dong-Son Hypothesis

As noted in the Introduction, this hypothesis supposes that SEA warp ikat weaving originated on the Asian mainland with the Dong-Son culture, a bronze-casting culture that was centered on the Red River delta area of what is now Vietnam, and that bronze items and drums were sources of motif and design inspiration for SEA weavers. The Dong-Son and neighboring cultures in Yunnan and Guangxi provinces of China made a wide variety of cast bronze items, but it was the drums that seem to have become a popular export item. In his review of Dong-Son finds, Bellwood [33] mentions 56 drums that have been found in various parts of the Indonesian archipelago. He notes that most were apparently made in the period after 100BC. The finds include two drums from Borneo, but none have been reported so far from the Philippines or Sulawesi.

**Figure 11:**
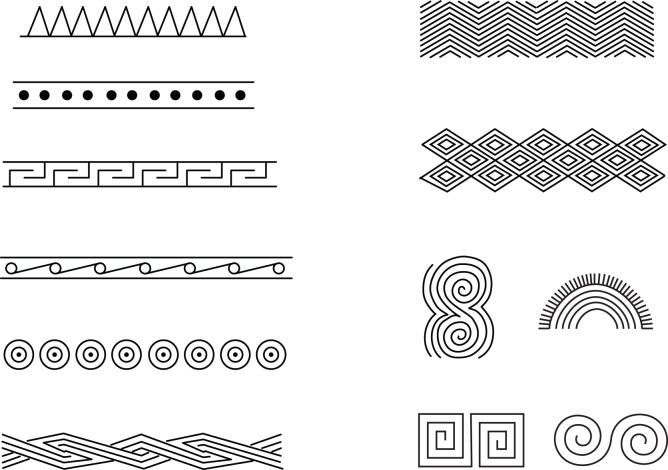
Geometric motifs from cast bronze drums from the Dong-Son culture.

If Dong-Son culture, and decorated bronze items in particular, were a source for ikat weaving motifs we would expect at least some of the ancestral motifs identified for the pSW and pAW traditions to be present on bronzes. The motifs on bronze artifacts fall into three categories: the first consists of realistic depictions of animals, including cranes, herons, peacocks, water buffalo, deer, frogs and other animals. The second category consists of stylized depictions of anthropomorphs with feathered headdresses, sometimes depicted on riverboats, and stylized bird motifs, in particular a flying heron. The third category consists of geometric repetitive motifs. The first two categories are not found in most SEA ikat, aside from the stylized flying heron that has been identified in Tai weft ikat by Howard [11]. Representative examples of the geometric category are shown in Fig.11. Comparing these motifs with the geometric motifs identified for the pSW and pAW there seems to be little or no overlap. In particular, the hook and rhomb motifs (Jnrd and Mnrd) that are ancestral to and characteristic of SEA weaving and that are often said to be ‘Dong-Son’ are not found on Dong-Son bronzes. Few, if any, of the other ancestral characters are shared in common. Casting the net somewhat wider and considering the entire set of ikat motifs, including characters from traditions near to Vietnam such as Hainan Island, it is hard to identify any that might be derived from Dong-Son bronzes. The shape of the Bayesian phylogenetic tree, with the prominent clade associated with Austronesian cultures, is also inconsistent with an origin centered in Vietnam. Neither can the Dong-Son hypothesis account for the presence of shared ikat weaving characters in regions such as the Philippines and Sulawesi where no Dong-Son artifacts have been found.

### 5. Discussion of Clades within the Phylogenetic Tree

This section compares the details of some of the groupings of taxa in the Bayesian tree and Neighbornet plot with existing understanding of the languages and weaving traditions. Some general themes that emerge from the detailed comparisons are discussed.

#### 5.1 The central flores and lamaholot groups

The Central Flores and Lamaholot weavers are from adjacent islands in the chain that extends from Java in the west to the Southern Maluku islands in the east. As noted, their traditions have been well studied by Barnes, Hamilton and others. Within the Lamaholot clade in the Bayesian tree there is a ‘core’ group of the weaving traditions of Lamalera, Atadei and Ili Api, which scholars have long recognized as constituting a closely related group, and that are geographically close to each other. The textiles of Alor and Solor also belong to this group, but seem to have branched off earlier in the line of descent. The textiles of Ili Mandiri show similarities with the Lamaholot group and are usually classed with this group, but the phylogenetic tree points to a more distant relationship, despite the Ili Mandiri weavers being geographically close to the core Lamaholot traditions. The Neighbornet plot suggests that the textiles of Ili Mandiri are the result (in part) of the horizontal exchange of design features with the neighboring central Flores group that lies just to the west.

Interestingly, the boundary between Central Flores and Lamaholot ikat weaving clades does not correspond precisely to linguistic boundaries. Linguists recognize the Lamaholot languages as a distinct group, but this group includes the Sikka language, whose textile tradition belongs to the Central Flores clade. The remaining Central Flores weavers speak languages classified in the Bima-Sumba group [92].

#### 5.2 Timor weaving traditions

The next island in the chain, to the east of Flores, is Timor. The phylogenetic tree divides weaving traditions on Timor into four sub-clades within the Austronesian clade. The Western Timor clade consists of the Ambenu, Amanuban, Amanatun and Miomafo traditions: all these weavers are speakers of dialects of the Uab Meto language. The Amarasi, who speak a more distantly related form of Uab Meto, occupy another clade. A third clade consists of Belu, Biboki and Insana weaving traditions. Biboki and Insana people also speak a variant of Uab Meto, but the Belu weavers have a distinct ethnic identity and speak the Tetun language. The Belu group are incomers who are believed to have arrived in the 14^th^ century [44], conquering the Malaka district and exercising political influence over the neighboring Biboki and Insana groups. It appears that one result of this incursion has been the replacement of native Biboki and Insana ikat weaving motifs by those of the dominant Belu group.

The fourth clade that is present on the main island of Timor is that of the Timor Lautem region at the far eastern tip of the island. Both the Neighbornet and Bayesian plots place this tradition within the Southern Maluku clade, though the Neighbornet plot also suggests that it has also borrowed characteristics from nearby Lamaholot weaving. This observation fits well with an intuitive assessment of cloth from this region, which is different in character from most other Timor weavings.

The Timor and Flores/Lamaholot regions illustrate a general point: there is some broad scale congruence between language and weaving traditions, but with differences at a local level, especially at the boundaries between language/weaving groups. This is perhaps to be expected since the method of transmission of language (from parent to child) is slightly different to the transmission of weaving (mother to daughter). As a result, correspondence between weaving and language may drift apart over the longer term, for example if weaving motifs but not language are spread by intermarriage or conscious copying of elite motifs between adjacent cultural groups.

#### 5.3 The central northern clade and the benua clade

**Figure 12:**
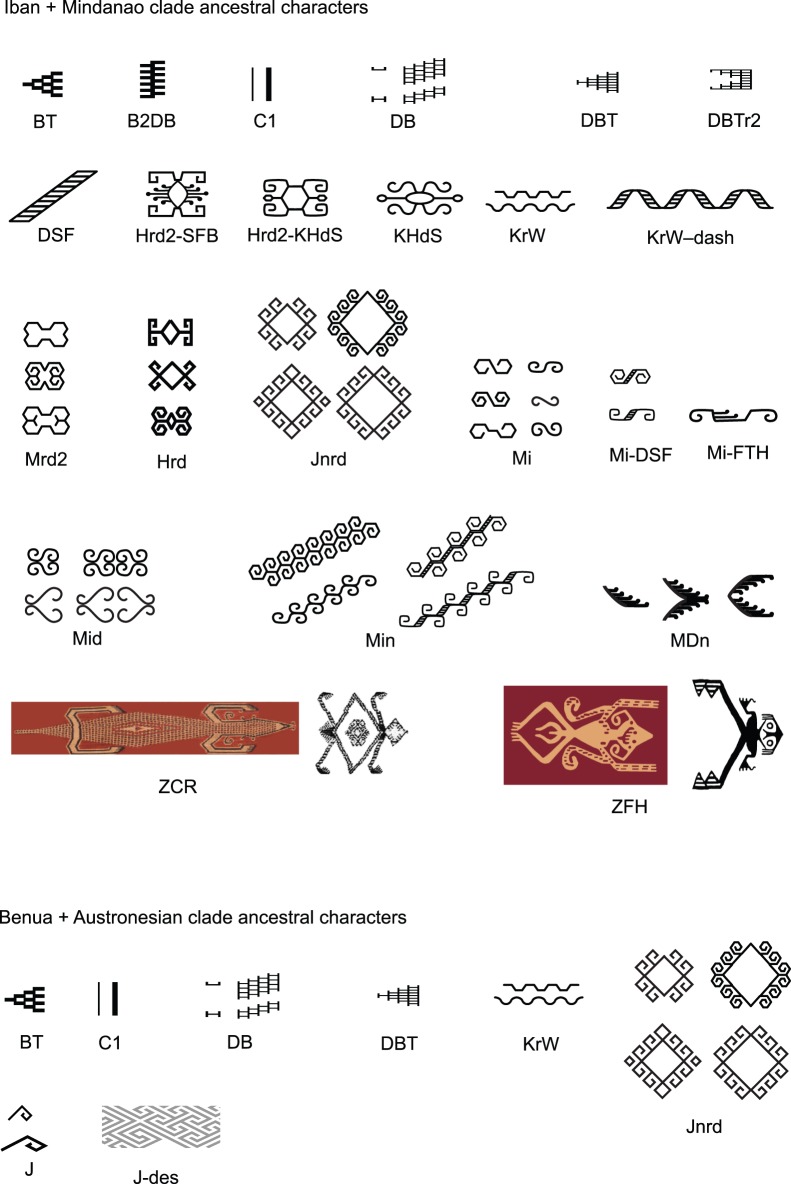
Reconstruction of the ancestral states (motifs) of the Iban-Mindanao clade and the Benua-Austronesian clade. The Iban and Mindanao weaving traditions form a sub-clade that has a rich group of 28 shared ancestral characters at its root, 25 of which are motifs (IM on Fig.7). In contrast the Iban-Mindanao and Benua traditions share a much smaller group of ancestral traditions at the root of the Benua-Austronesian clade (BA on Fig.7), despite the closer geographic proximity of the Iban and Benua weavers in Borneo.

Despite being separated by considerable geographic distance (and a sea crossing), the Iban and Mindanao traditions form a distinct and well-supported clade, sharing 28 ancestral characters. In contrast, the Benua weaving tradition, which is geographically close to the Iban weavers, occupies another clade entirely and shares only a basal subset of ancestral characters with the Iban-Mindanao clade, reflecting an early divergence near to the root of the phylogenetic tree. Ancestral motifs at the relevant points in the phylogenetic tree (IM and BA in Fig.7) are shown in Fig.12, highlighting the similarities and differences. This finding is both surprising and interesting. In a qualitative sense, Iban and Benua textiles share some superficial similarities (both groups produce warp ikat textiles with dominant red-brown coloring) but the small number of characters that they share belies this appearance. Both groups speak Austronesian languages belonging to the Malayo-Polynesian group. The culture and languages of these groups deserve more study to try to understand how they come to have such widely divergent cultural traditions. Similarly, the connection between Iban and Mindanao weaving traditions is closer than expected, given the geographic and linguistic distances separating them. The Iban group has a tradition of migration and may have moved over considerable distances in the past.

#### 5.4 The daic clade

This clade includes the mainland Tai and the Hainan Meifu Li weaving traditions. As noted in the Introduction, the Meifu Li tradition is of key importance in understanding non-Austronesian ikat weaving and hence in defining the common ancestral tradition. The Bayesian tree shows that Meifu Li weaving is most closely related to Tai weaving and is not a sub-clade of Austronesian weaving, which parallels the linguist’s understanding of the relationship between Tai and Li languages and Austronesian. The phylogenetic analysis also confirms the qualitative impression of Meifu Li ikat textiles (Fig.3). At first sight their Southeast Asian character is apparent, but detailed analysis of characters reveals that the Meifu Li tradition is actually highly divergent from neighboring Austronesian weaving and branched off from their common ancestor at an early date (perhaps before 4500 BP). Meifu Li ikat motifs also lack any discernable influence from Indian trade textiles, which makes them a useful comparison tool for identifying this influence in their neighbors.

### 6. Geographical Distribution of Warp Ikat Weaving

A striking feature of the geographic distribution of warp ikat weaving traditions in the Indonesian archipelago is the high density of warp-ikat based traditions within eastern part of the Indonesian archipelago (Figs.8 and 9) and their virtual absence to the west of this region. In the western part of the archipelago, most woven textiles are decorated using weft-based techniques (supplementary weft, weft ikat). The division between these regions, which runs through the western edge of Flores, lies somewhat to the east of a major linguistic division between the Central Malayo-Polynesian (CMP) languages of Flores, Sumba and the islands to the east, and the Western Malayo-Polynesian (WMP) languages of Java, Sumatra, Borneo and the Malay peninsula. It also corresponds, in approximate terms, to the limits of influence of Indianized cultures centered on Java and Sumatra, during the last 1000 years. It remains an open question as to which of these factors, working singly or in combination, accounts for this prominent cultural division.

### 7. Comparison with Other Traditional Technologies

As noted in the Introduction, loom weaving is part of a group of technologies including bark-cloth making, pottery and the making of high quality stone tools, that is associated with Austronesian migrations in ISEA. In each of these cases several distinct processes have accompanied the process of dispersal of these novel technologies. Green identified these in his Triple-I model [93], comprising *intrusion* followed by *innovation* and *integration*. Examples include the development and dispersal of Lapita decorated pottery and Oceanic bark-cloth, as well as the ikat-decorated weaving discussed here. A common factor in each of these dispersals has been innovation associated with the arrival of these skills in new in new locations, a process that a biologist might call niche expansion. This process has been responsible for a significant part of the diversity of ikat weaving in the ISEA region today.

To the list that Green defined, we might also add ‘loss’, since both loom weaving and pottery, unlike bark-cloth, did not spread through the entire range of Austronesian expansion and are not practiced by all groups even within their ranges. This is perhaps due to the greater complexity and more exacting raw-material requirements of both weaving and pottery. This may have made them more susceptible to population bottlenecks and founder-effects, particularly in colonization events in the more remote islands of the Oceanic region. It also provided the impetus for some groups to specialize in these skills, and to trade the resulting goods with their neighbors, a factor that has played a role in the geographic distribution of weaving in the Indonesian archipelago.

### Conclusions

In recent decades it has become well accepted that some aspects of human culture evolve by processes similar to biological evolution, especially activities passed from parent to child such as weaving. This intergenerational mode of transmission makes it possible, in principle, to deduce phylogenetic trees representing the descent of these traditions. In this study, such a tree has been derived for the case of warp ikat weaving in Southeast Asia, from a database of characters based on motifs and design characteristics, using Bayesian MCMC methods. The Neighbornet plot provides a useful model-independent check that the groupings identified in this analysis have a sound basis. The results support some long-held views amongst textile specialists and anthropologists that these weaving traditions have a common origin, as well as providing some new details and a working taxonomy. The main conclusions can be summarized as follows:

1. The Southeast Asian warp ikat weaving traditions are related, and share a common ancestor amongst neolithic cultures on the Asian mainland.
2. The phylogenetic tree and geographic dispersal pattern for warp ikat in ISEA is similar to that deduced for the migration of Austronesian peoples in the region. This is most easily explained if Austronesian migrants introduced warp ikat into ISEA, along with other technologies.
3. Analysis of ancestral states and tree topology show that it is unlikely that ISEA warp ikat motifs and weaving originated with Bronze Age Dong-Son culture.

The ikat-decorated textiles of Southeast Asia exhibit a complex set of characteristics, including both shared features and variation. Analysis of these characteristics using statistical tools enables a new appreciation of both the variety of these textiles and of their underlying unity.

## Supporting Information

Data S1 Table of data used in this study.
(PDF)

Motifs S1 Description of coding system used for warp ikat motifs and examples of motifs and primitives.
(PDF)

TaxaChars S1 Definitions of taxa (weaving traditions) and characters used in this study.
(PDF)
