# Supplementary material for: Investigating Cultural Evolution Using Phylogenetic Analysis: The Origins and Descent of the Southeast Asian Tradition of Warp Ikat Weaving
Source: PLoS One. 2012 Dec 18;7(12):e52064. doi: 10.1371/journal.pone.0052064 (PMC3525544; doi:10.1371/journal.pone.0052064)
Supplement: Motifs S1 — Description of coding system used for warp ikat motifs and examples of motifs and primitives. (PDF) [file pone.0052064.s002.pdf]

Classification and coding of warp ikat motifs: Introduction

[Supplemental material for the paper *Investigating cultural evolution using phylogenetic analysis: the origins and descent of the Southeast Asian tradition of warp ikat weaving* by Christopher D Buckley.]

This classification and coding system was developed specifically for use with warp ikat, defined as designs that are created on the warp fibers of a fabric prior to weaving, using a resist technique.

Ikat weavers assemble complex geometric motifs by repeating simple shapes (‘primitives’) and applying transformations to them (reflection, rotation, glide). This means that motifs can be classified and systematized by identifying the primitives that they are composed of and the symmetry operations that are used to produce the motifs. Classifying motifs in this way is important for two reasons. Firstly, it enables motifs to be identified that look superficially different but are actually closely related. Secondly, it allows superficially similar motifs that are actually composed of different primitives (and thus are different from a weaver’s perspective) to be eliminated.

Warp versus weft axis

Weaving geometry differs from regular Cartesian plane geometry in one important respect: the two axes (x,y in the Cartesian plane, weft, warp in the weaving plane) are not equivalent on woven textiles. As an illustration of this consider these two simple shapes, which are equivalent in a Cartesian sense but different from a weaver’s perspective (imagining the warp lying in the same direction as this line of text):

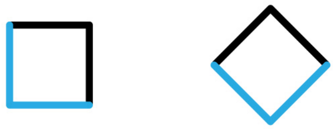

The basic shape that must be tied on the warp (shown in color) is different: in one case the weaver must tie resist knots along a row of warp threads and then along several bunches of warp threads lying at right angles. In the other case, it involves tying two diagonal rows of knots on the ikat frame. Another way of looking at this is that an older weaver must teach a younger weaver a different procedure for making these two motifs, and that the ‘instruction’ that is passed from one generation to another is different.

In the classification system, primitives are labeled A, B, C etc, in approximate order of increasing complexity. They are shown in color on the left hand side of the following pages. The motifs that are made up from them are given unique labels based on the symmetry operations used to create them:
r = reflection in weft axis
d = reflection in warp axis
i = rotation through 180 degrees
n = glide

Motifs that are composed of two or more simpler motifs are indicated by hyphens, for example, Ed-ITSN is the star shape ITSN enclosed by brackets Ed. This motif is found on textiles from Kisar and neighboring islands.

This labeling system is used in a semi-systematic way to differentiate motifs, since a fully systematic nomenclature would be too cumbersome for practical use, particularly with more complex motifs.

In many cases this approach reveals that motifs that look superficially different are actually similar from a weaver's perspective, because they are made up of similar shapes. For example the distinction between the "line" form of a motif and the "block" (filled in) is generally of no great significance to a weaver tying ikat resists, since the shape that must be remembered is the same in the first instance, even though the final result looks different. Duplication by reflection in the warp axis is also a trivial matter for the weaver, since in most cases this can be done by tying several two (or more) sets of warp bundles together and then separating them into mirror image halves after tying and dyeing. These features explain why (for example) the group of simple motifs labeled Gr and Grd, some of which appear different at first sight are actually very similar as far as weavers are concerned, and often appear together in the same textile or in similar textiles:

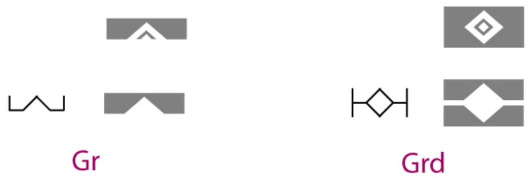

Van Vuuren drew attention to this phenomenon in her account of interviewing Tanimbar weavers [12]. She noted that they paid little attention to the shapes that she recognized as 'motifs' on finished textiles, instead preferring to discuss the shapes that they needed to remember when tying resists onto the warp.

Not all of the differences in primitives correspond to meaningful differences from a weaver's point of view. For example, in many cases square curls, hexagonal shaped curls and smoothly curved curls are essentially equivalent. For this reason motifs that occur interchangeably on textiles of a tradition or closely related traditions are grouped together into a single character, since the differences between them convey no useful information. This has been done on a pragmatic basis, based on analysis of a large set of textiles and observation of weavers in the field.

A degree of familiarity with weaving motifs and textiles generally is required in order to make these judgments and others like them, and the challenges involved are quite analogous to the decisions that must be made when coding linguistic data or morphological characteristics in biology.

Conservatism and copying in warp ikat decoration
As mentioned, weavers use a limited vocabulary of primitives to construct motifs. These are simple shapes that are used in building-block fashion to construct more complex motifs. The use of this vocabulary and the conservative nature of most traditional weaving is particularly apparent in cases where warp ikat weavers have consciously copied motifs from external sources. The cloths most commonly imitated by Indonesian weavers in the past few centuries have been Indian imported silk patola (made using a complex double ikat technique), and the Indian printed cotton trade cloths that were themselves imitations of patola designs. These cloths had high status amongst Indonesian families and were often used as heirloom and bridewealth items, and their designs were sometimes copied into locally made warp ikat. For example, the biboek'sa motif of the Biboki weavers of West Timor has been correctly identified by Barrkman [46] as a patola-inspired creation, with the characteristic patola features of white designs outlined in black on a dark red ground. The biboek'sa motif successfully evokes patola textiles and includes some innovations in layout and coloring, but, remarkably, the motif itself has been re-constructed from primitives that were already familiar to Biboki weavers. It has also been modified from an all-over design to a linear format suited to motifs organized into bands in Biboki sarongs:

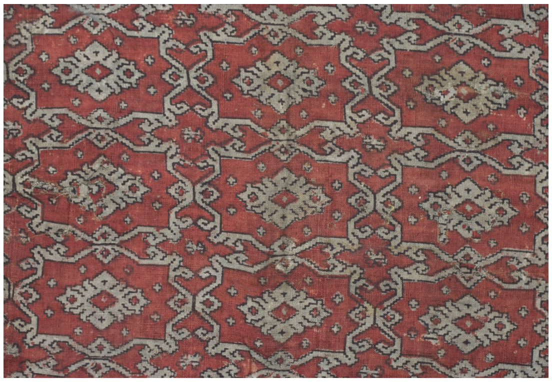
Fragment of Indian printed cotton trade cloth, 19th century (detail)

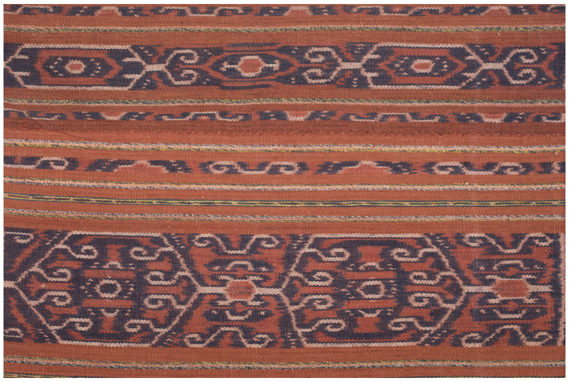
Contemporary Biboki sarong with biboek'sa design, based on Indian trade cloths

A rough impression of the overall shape of the Indian motif is recognizable in the Biboki sarong, together with the floral feature at the center and presence of hooks, but the Biboki version is actually composed of hook-and-rhomb shapes that were familiar to the Biboki weavers. The original Indian design falls outside the classification system for warp ikat presented here since it is composed of entirely different shapes, but the Biboki version is classifiable as the motif Mnrd-IN, which is grouped with other similar motifs into the character JnrdIN.

Barnes, in her classic study of Lamaholot textiles [4], also reviewed a number of instances of motifs that were said to be "patola" derived. She found that in most cases these had also been re-interpreted by Lamaholot weavers, to the extent that she found it difficult to identify the Indian prototypes, so thoroughly had they been assimilated into local tradition.

Motifs
The following pages show examples of motifs from warp ikat textiles. Drawings are by the author.

Geometric motifs
The column on the right lists primitives, the basic shapes from which motifs are composed. The main section shows motifs derived from those primitives.

(references: refer to main paper)

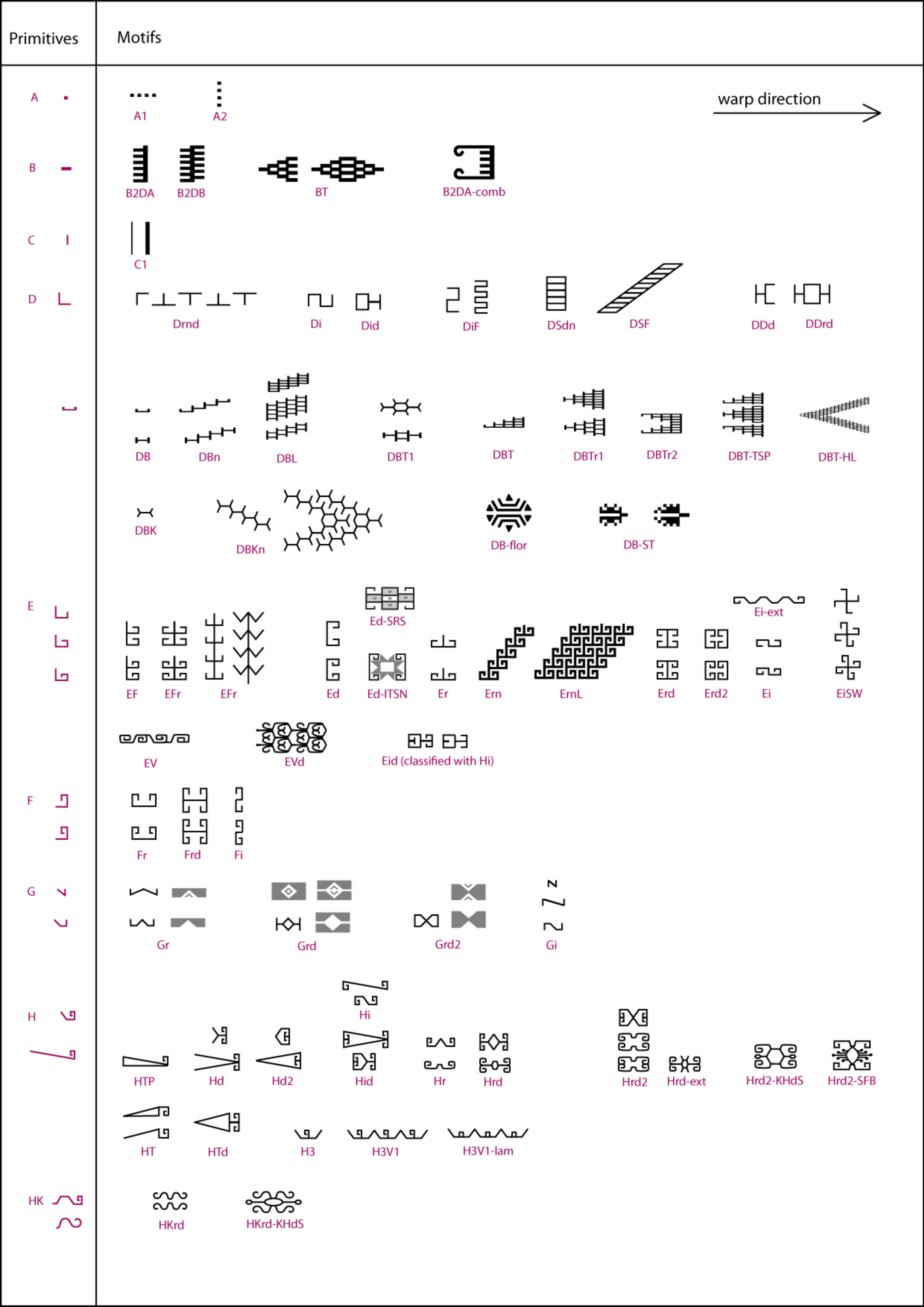
Primitives
Motifs
A
A1
A2
warp direction
B
B2DA
B2DB
BT
B2DA-comb
C
C1
D
Drnd
Di
Did
DiF
DSdn
DSF
DDd
DDrd
DB
DBn
DBL
DBT1
DBT
DBTr1
DBTr2
DBT-TSP
DBT-HL
DBK
DBKn
DB-flor
DB-ST
E
Ed-SRS
Ei-ext
EF
EFr
EFr
Ed
Ed-ITSN
Er
Ern
ErnL
Erd
Erd2
Ei
EiSW
EV
EVd
Eid (classified with Hi)
F
Fr
Frd
Fi
G
Gr
Grd
Grd2
Gi
H
Hi
HTP
Hd
Hd2
Hid
Hr
Hrd
Hrd2
Hrd-ext
Hrd2-KHdS
Hrd2-SFB
HT
HTd
H3
H3V1
H3V1-lam
HK
HKrd
HKrd-KHdS

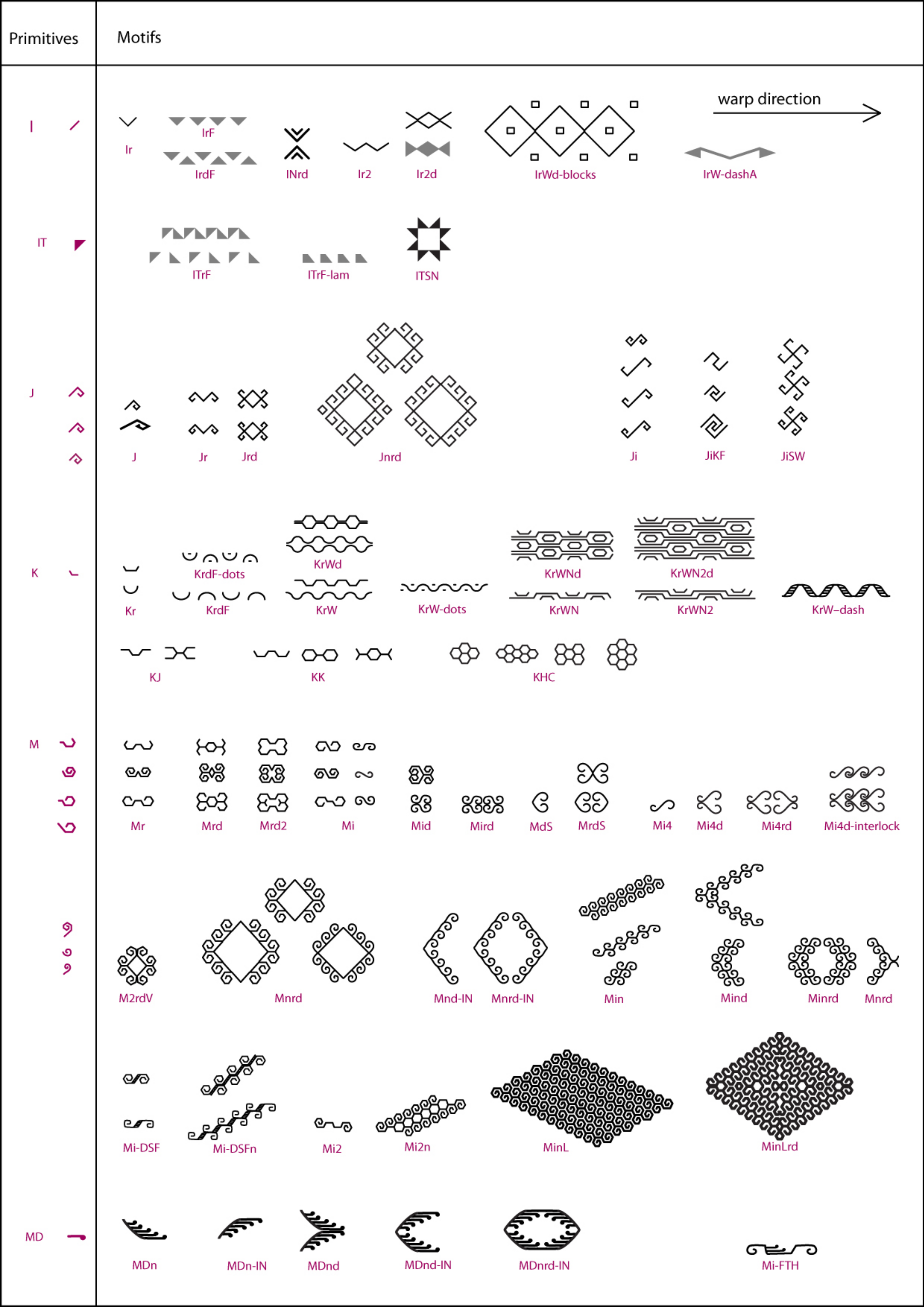

Primitives
Motifs
warp direction
I
Ir
IrF
IrdF
INrd
Ir2
Ir2d
IrWd-blocks
IrW-dashA
IT
ITrF
ITrF-lam
ITSN
J
J
Jr
Jrd
Jnrd
Ji
JiKF
JiSW
K
Kr
KrdF-dots
KrdF
KrWd
KrW
KrW-dots
KrWNd
KrWN
KrWN2d
KrWN2
KrW–dash
KJ
KK
KHC
M
Mr
Mrd
Mrd2
Mi
Mid
Mird
MdS
MrdS
Mi4
Mi4d
Mi4rd
Mi4d-interlock
M2rdV
Mnrd
Mnd-IN
Mnrd-IN
Min
Mind
Minrd
Mnrd
Mi-DSF
Mi-DSFn
Mi2
Mi2n
MinL
MinLrd
MD
MDn
MDn-IN
MDnd
MDnd-IN
MDnrd-IN
Mi-FTH

warp direction →

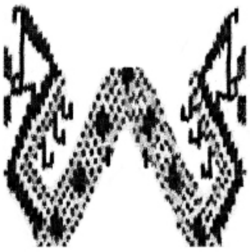

ZNAG
(Tai Daeng weft ikat, after [11] pl.27)

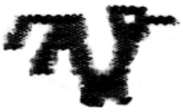

ZBHC
(Amanuban, author's coll KT88)

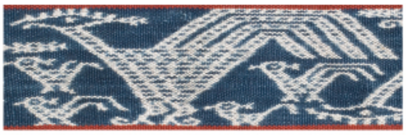

ZBHC
(Insana, author's coll KT112)

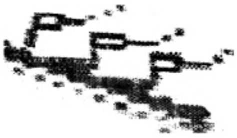

ZHhead
(Tai Daeng, author's coll KT80)

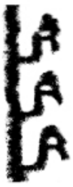

ZHhead
(Lampung, after [2] p268)

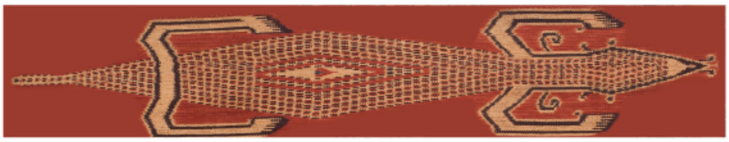

ZCR
(Iban, author's coll. KT51

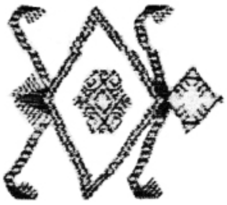

ZCR
(Mindanao, Mandaya after [57] fig.1.54)

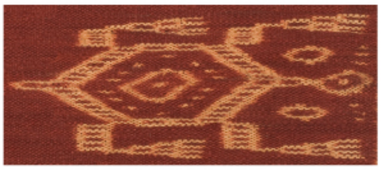

ZCRTL
(Belu, author's coll. KT28)

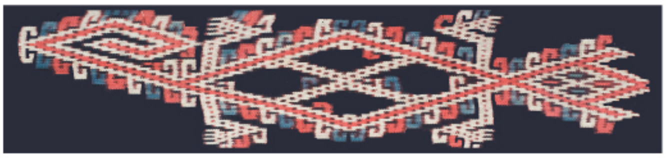

ZCSRT
(Amanuban, author's coll. KT88)

warp direction →

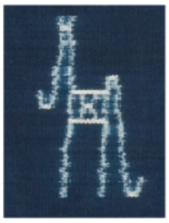

ZEQ
(Ngada, author's coll. KT35)

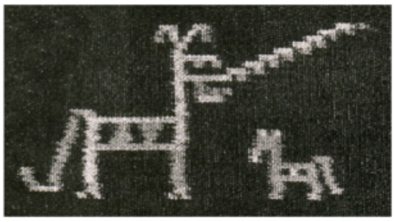

ZEQ
(Ngada, after [8] fig.5-17)

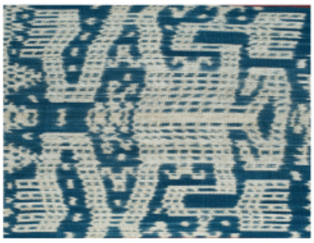

ZFH
(Amanatun, author's coll. KT90)

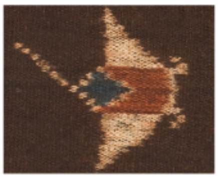

ZFMR
(Lamalera, author's coll. KT16)

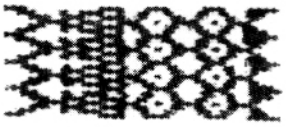

ZTF
(Tanimbar, Yamdena, after [55] p37 fig.17)

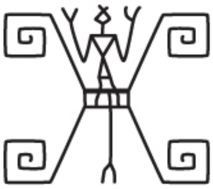

ZHG
(Ngada, after [66] 46)

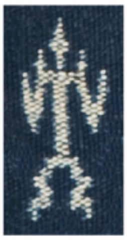

ZHPT
(Hainan Meifu Li, author's coll. CET365)

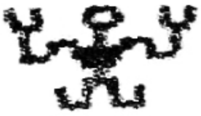

ZHSTP
(Babar, author's coll. CL2)

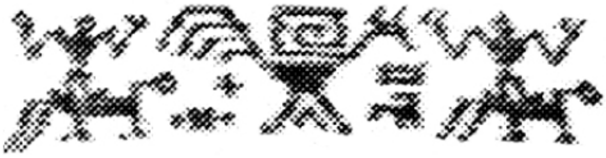

ZHSB
(Luang, after [64] 543)

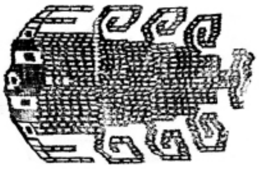

WB
(Amanatun, after [44] pl.51)

Textile layout types/ design features

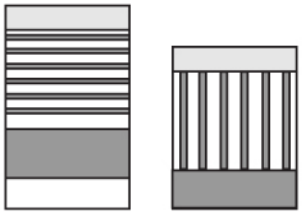

asymTS
asymmetric
one or more panels plus waistband

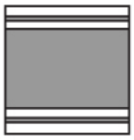

SSB
symmetric
one panel

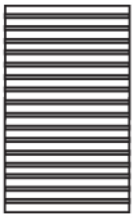

STA
symmetric
two or more panels

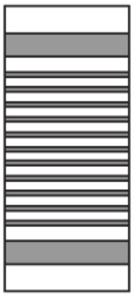

STB
symmetric
two or more panels

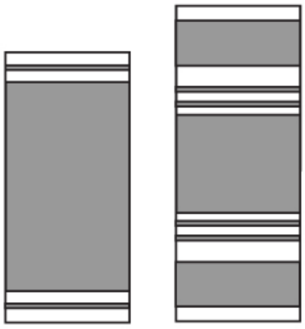

STC
symmetric
two or more panels

Other design/layout features
(motifs shown are intended to illustrate design features rather than to represent particular motifs)

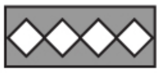

rhomb
motifs organized into diamond shaped panels

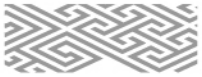

J-des
motifs composed of semi-abstract rhombic hook shapes

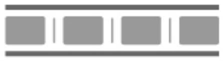

pan
motifs divided into panels separated by dividing bands

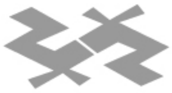

i-des
motifs with 2-fold rotational symmetry (only)
