# Supplementary material for: Investigating Cultural Evolution Using Phylogenetic Analysis: The Origins and Descent of the Southeast Asian Tradition of Warp Ikat Weaving
Source: PLoS One. 2012 Dec 18;7(12):e52064. doi: 10.1371/journal.pone.0052064 (PMC3525544; doi:10.1371/journal.pone.0052064)
Supplement: TaxaChars S1 — Definitions of taxa (weaving traditions) and characters used in this study. (PDF) [file pone.0052064.s003.pdf]

Supplemental Material: Definitions of Taxa and Characters
[Supplemental material for the paper *Investigating cultural evolution using phylogenetic analysis: the origins and descent of the Southeast Asian tradition of warp ikat weaving* by Christopher D Buckley.]

1) Definitions of Weaving Taxa

**Weaving tradition (taxon)**	**Definition**	**Language(s)**	**Language classification** (World Atlas of Languages)	**Language classification** (Ethnologue)
Tai (Weft Ikat)	Textiles made using weft ikat by Tai Dam, Tai Daeng, Tai Dam, Tai Mai Chau, Tai Waat, Tai Khang and related groups the regions around Muang Phan and Xam Nuea (borders of Laos and Northern Vietnam). These weavings seem to be a transposition of older warp ikat traditions with relatively little Indian influence (outgroup).	Tai Dam<br>Tai Daeng and related dialects	Tai-Kadai	Tai-Kadai, Kam-Tai
Hainan Meifu Li	Warp ikat tubeskirts made by the Meifu Li subgroup of the Li people of Hainan Island, politically part of China.	Hlai	Tai-Kadai	Tai-Kadai
Sumatra Lampung	Tapis inuh (tubeskirts) from Liwa region at the southern tip of Sumatra, made with warp ikat bands interspersed with embroidery, single-color type)	Lampung	Lampungic	MP
Philippines Mindanao	Warp ikat textiles, mainly tubeskirts and ceremonial hangings, made by T'boli, Kulaman, Bagobo, B'laan and Mandaya groups in southern Mindanao.	1) T'boli, B'laan, Bagobo,<br><br>2) Mandaya	South Mindanao	1) MP, South Mindanao<br><br>2) MP, Greater Central Philippine
Borneo Iban	Warp ikat textiles, including tubeskirts and ceremonial hangings (pua kumbu) made by Iban people in the Upper Kapuas River region of Kalimantan and adjacent regions in Borneo	Iban	Malayic	MP, Malayic
Borneo Benua	Warp ikat textiles, mainly wrap-around skirts made from ulap doyo (local bast fiber) by Benua people in the Kutai region of Kalimantan	Lawangan	Malayic	MP, Barito
Flores Ende	Warp ikat textiles, mainly tubeskirts and men's wraps, made in central Flores in Ende, Ndona and western part of Wolowaru district, including Onelako.	Ende-Lio	CMP	CMP, Bima-Sumba
Flores Lio	Warp ikat textiles, mainly tubeskirts and men's wraps made in central Flores in Nila	Ende-Lio	CMP	CMP, Bima-Sumba

	and areas to the east, Nggela and Wolojita.			
Flores Sikka	Warp ikat textiles, mainly tubeskirts and men's wraps made in Sikka areas including coastal Sikka Natar styles and inland weaving groups. Two different styles (Sikka Natar and inland Iwang Gete) are sometimes recognised [], though both are made by the same weavers and are not distinguished here.	Sikka	CMP	CMP, Timor, Flores-Lembata
Flores Ngada	Warp ikat textiles, mainly tubeskirts and men's wraps made by Ngada peoples in central Flores	Ngada	CMP	CMP, Bima-Sumba
Sumba East	Warp ikat textiles (tubeskirts and men's ceremonial wraps called hinggi) made in the eastern part of Sumba.	Bima	CMP	CMP, Bima-Sumba
Roti	Warp ikat textiles (tubeskirts and men's wraps) made on the island of Roti (Rote), near the western tip of Timor.	Bilba	CMP	CMP, Timor, Nuclear Timor, West
Savu	Warp ikat textiles (tubeskirts and men's wraps) made on the island of Savu.	Savu	CMP	CMP, Bima-Sumba
Lamaholot Ili Mandiri	Warp ikat textiles (mainly tubeskirts) made in the Ili Mandiri region of eastern Flores	Lamaholot	CMP	CMP, Timor, Flores-Lembata
Lamaholot Solor	Warp ikat textiles (mainly tubeskirts) made on the island of Solor (excluding Tanalein and Lewokukung districts)	Lamaholot	CMP	CMP, Timor, Flores-Lembata
Lamaholot Lamalera	Warp ikat textiles (mainly tubeskirts) made in the village of Lamalera in eastern Flores	Lamaholot	CMP	CMP, Timor, Flores-Lembata
Lamaholot Atadei (Lerek Peninsula)	Warp ikat textiles (mainly tubeskirts) made in the village of Atadei in eastern Flores	Lamaholot	CMP	CMP, Timor, Flores-Lembata
Lamaholot Ili Api	Warp ikat textiles (mainly tubeskirts) made in the Ili Api district in eastern Flores	Lamaholot	CMP	CMP, Timor, Flores-Lembata
Alor	Warp ikat textiles (mainly tubeskirts) made on the island of Alor (northwestern, Kalabahi area, see BKM91 p214)	Alor (several Trans-New Guinea languages also spoken nearby)	Timor-Alor-Pantar	CMP, Timor, Flores-Lembata
Timor Amarasi (excluding Baun textiles)	Warp ikat textiles (tubeskirts and men's wraps) made by Amarasi people at the western end of Timor, inland from Kupang. Excludes types characterised as "Baun" textiles.	Amarasi (similar to Uab Meto)	CMP, Timorese	CMP, Timor, Extra-Ramelaic, West
Timor Amanatun	Warp ikat textiles (tubeskirts and men's wraps) made in the region of western Timor that	Uab Meto	CMP, Timorese	CMP, Timor, Extra-Ramelaic, West

	corresponds to the former Amanatun and Nenometan kingdoms [TWTp113], including Oinlasi.			
Timor Amanuban	Warp ikat textiles (tubeskirts and men's wraps made in the region of western Timor that corresponds to the former Amanuban kingdom, including Soe and Niki-Niki.	Uab Meto	CMP, Timorese	CMP, Timor, Extra-Ramelaic, West
Timor Miomafo	Warp ikat textiles (tubeskirts and men's wraps made in the region of western Timor that corresponds to the former Miomafo kingdom, including Kefamenanu (bordering Biboki kingdom).	Uab Meto	CMP, Timorese	CMP, Timor, Extra-Ramelaic, West
Timor Ambenu (East Timor enclave)	Warp ikat textiles (tubeskirts and men's wraps made in the area of western Timor that is now an enclave of East Timor, corresponding to the former Miomafo kingdom.	Uab Meto	CMP, Timorese	CMP, Timor, Extra-Ramelaic, West
Timor Insana	Warp ikat textiles (tubeskirts and men's wraps made in the region of central Timor (politically West Timor) that corresponds to the former Insana kingdom, including Maubesi and Oelolok.	Uab Meto	CMP, Timorese	CMP, Timor, Extra-Ramelaic, West
Timor Biboki	Warp ikat textiles (tubeskirts and men's wraps made in the region of central Timor (politically West Timor) that corresponds to the former Biboki kingdom.	Uab Meto	CMP, Timorese	CMP, Timor, Extra-Ramelaic, West
Timor Malaka (Belu)	Warp ikat textiles (tubeskirts and men's wraps made in the region of central Timor (politically part of West Timor) that corresponds to the Malaka district of the former Belu kingdom.	Tetun	CMP	CMP, Timor, Extra-Ramelaic, Central
Timor Lautem	Warp ikat textiles (tubeskirts and men's wraps) from Los Palos and nearby districts at the far eastern tip of East Timor.	Fataluku	Timor-Alor-Pantar	Trans-New Guinea, West, East Timor, Fataluku-Oirata
Kisar	Warp ikat textiles (tubeskirts, men's wraps and loincloths) made on the island of Kisar.	1) Kisar<br><br>2) Oirata	CMP<br><br>TNG	CMP, Timor, Extra-Ramelaic, East, Luang-Kisar
Luang	Warp ikat textiles (tubeskirts, men's wraps and loincloths) made on the island of Luang.	Luang	CMP	CMP, Timor, Extra-Ramelaic, East, Luang-Kisar
Babar - Masela	Warp ikat textiles (tubeskirts, men's wraps and loincloths) made on the island of Masela,	Babar-South	CMP	CMP

	part of the Babar archipelago.			
Babar - Wetan	Warp ikat textiles (tubeskirts, men's wraps and loincloths) made on the island of Wetan, part of the Babar archipelago.	Babar-North	CMP	CMP
Tanimbar - Selaru	Warp ikat textiles (tubeskirts, men's wraps and loincloths) made on the island of Selaru, part of the Tanimbar archipelago.	Selaru	CMP	CMP, Southeast Maluku
Tanimbar - Yamdena	Warp ikat textiles (tubeskirts, men's wraps and loincloths) made in the area near the town of Yamdena, on the central island of the Tanimbar archipelago.	Yamdena	CMP	CMP, Southeast Maluku
Tanimbar - Larat	Warp ikat textiles (tubeskirts, men's wraps and loincloths) made on the island of Larat, part of the Tanimbar archipelago.	Fordata	CMP	CMP, Southeast Maluku
Sulawesi Toraja	Warp ikat textiles, mainly tubeskirts and large ceremonial hangings, made in the Kalumpang and Toraja districts of Sulawesi, by Toraja people. These districts may have had distinctive styles, but widespread trade between them makes distinguishing point of origin difficult and they are combined here.	Toraja	Sulawesi	MP, South Sulawesi

Language definitions are from the World Atlas of Language Structures by Dyer et al, and Ethnologue:

Dryer, Matthew S. & Haspelmath, Martin (eds.). 2011. The World Atlas of Language Structures Online. Munich: Max Planck Digital Library. Available online at http://wals.info/ Accessed on 2012-07-16.

Lewis, M. Paul (ed.), 2009. Ethnologue: Languages of the World, Sixteenth edition. Dallas, Tex.: SIL International. Online version: http://www.ethnologue.com/

2) **Definitions of Characters**

The column “Character name” lists the characters included in the dataset. The column “Included Motifs” shows which motifs are included in the definition of this character. In some cases one character corresponds to an individual motif, in other cases several variants are listed. These are variations that are similar to the degree that they can be regarded as the same motif, for example angular and rounded forms of the same shape, or variations made by trivial repetitions of the basic shape. For example, ikat weavers will commonly tie a bundle of threads with a design, then separate the bundle after dyeing into two or more sets (either identical or mirror-image versions), producing a repeating design in a similar way that a folded paper-cut will make a repeating design. The motifs (including variant forms) are shown in the supplementary file MotifsS2.

**Character name**	**Character number**	**Included Motifs**
A2	1	A2
BT	2	BT
B2DA	3	B2DA
B2DB	4	B2DB
B2DA-comb	5	B2DA-comb
C1	6	C1
DiF	7	DiF
Drnd	8	Drnd
DB	9	DB and variants
DBT1	10	DBT1
DBT	11	DBT, DBTr1
DBT-HL	12	DBT-HL
DBTr2	13	DBTr2
Gd-c	14	Gd-c
Gd-cd	15	Gd-cd
DBST	16	DBST, Ed-DBST
DBT-TSP	17	DBT-TSP
DBKn	18	DBKn
DB-flor	19	DB-flor
DSdn	20	DSdn
DSF	21	DSF
DDrd	22	DDrd
Ed-SRS	23	Ed-SRS
Ed-ITSN	24	Ed-ITSN
Efr	25	Efr or Mr variation
EiSW	26	EiSW
Er	27	Er, Erd, Erd2
Ern	28	Ern, ErnL
EF	29	EF
Frd	30	Frd
Fi	31	Fi
Gr	32	Gr, Gi
Grd	33	Grd
Grd2	34	Grd2
HT	35	HT, HTP
Hd	36	Hd
Hd2	37	Hd2
HTd	38	HTd
Hi	39	Hi, Hid (elongated and hooks attached to triangular shapes only, others classified under Ei and Eid)
Hrd2	40	Hrd2
Hrd2-SFB	41	Hrd2-SFB

H3	42	H3
H3V1-lam	43	H3V1-lam
Ir2d	44	Ir2d
IrdF	45	IrdF
ITrFlam	46	ITrF-lam
ITrF	47	ITrF
IrWdashA	48	IrW-dashA
INrd	49	INrd
IrWdblock	50	IrWd-blocks
ITSN	51	ITSN
KJ	52	KJ, KK and combinations
KHdS	53	KHdS, Hrd2-KHdS
HKrdKHdS	54	HKrd-KHdS
KrdF	55	KrdF
KrdFdots	56	KrdF-dots
KrW	57	KrW, KrWd
KrWdots	58	KrW-dots
KrWN	59	KrWN, KrWNd, KrWN2, KrWN2d
KrWdash	60	KrW-dash
KHC	61	KHC
Mrd2	62	Mrd2
Mr2rd	63	Mr2rd
Hrd	64	Hrd, Jrd, Mrd
Hrdext	65	Hrd-ext
Jnrd	66	Jnrd, Mnrd (and variant JnrdV)
Jnrddb	67	Jnrd-dashed band
JnrdIN	68	Jnrd-IN, Mnrd-IN
J	69	J
Jdes	70	J-des
Di	71	Di
Ei	72	Ei and variations
EiExtd	73	Ei-extdash
Mi	74	Mi
MiDSF	75	Mi-DSF
MiFTH	76	Mi-FTH
Mid	77	Mid, Mird, Mi4d, Mi4rd
Md	78	MdS, MrdS
Min	79	Min, Mind, Minrd (including DSF versions)
Mi2	80	Mi2, Mi2n
MDn	81	MDn, MDnd, MDnrd, MDnd-IN, MDnrd-IN
ZNAG	82	ZNAG
ZBHC	83	ZBHC
ZHhead	84	ZHhead
ZCR	85	ZCR
ZCRTL	86	ZCRTL
ZCSRT	87	ZCSRT
ZEQ	88	ZEQ
ZHEQ	89	ZHEQ
ZFH	90	ZFH
ZFMR	91	ZFMR
ZKBF	92	ZKBF
ZTF	93	ZTF
ZSTG	94	ZSTG
ZHG	95	ZHG
ZHPT	96	ZHPT
ZHSTP	97	ZHSTP
ZHSB	98	ZHSB
ZWB	99	ZWB

rhomb	100	rhomb
pan	101	pan
i-des	102	i-des
asymTS	103	asymTS
STA	104	STA
STB	105	STB
STC	106	STC
SSA	107	SSA
SSB	108	SSB
CBR	109	CBR
SBL	110	SBL
